# DELLA-mediated PIF degradation contributes to coordination of light and gibberellin signalling in *Arabidopsis*

**DOI:** 10.1038/ncomms11868

**Published:** 2016-06-10

**Authors:** Kunlun Li, Renbo Yu, Liu-Min Fan, Ning Wei, Haodong Chen, Xing Wang Deng

**Affiliations:** 1 State Key Laboratory of Protein and Plant Gene Research, Peking-Tsinghua Center for Life Sciences, School of Advanced Agriculture Sciences, and School of Life Sciences, Peking University, Beijing 100871, China; 2 Department of Molecular, Cellular and Developmental Biology, Yale University, New Haven, Connecticut 06520, USA

## Abstract

Light and gibberellins (GAs) antagonistically regulate hypocotyl elongation in plants. It has been demonstrated that DELLAs, which are negative regulators of GA signalling, inhibit phytochrome-interacting factors 3 and 4 (PIF3 and PIF4) by sequestering their DNA-recognition domains. However, it is unclear whether there are other mechanisms of regulatory crosstalk between DELLAs and PIFs. Here, we demonstrate that DELLAs negatively regulate the abundance of four PIF proteins through the ubiquitin–proteasome system. Reduction of PIF3 protein abundance by DELLAs correlates closely with reduced hypocotyl elongation. Both sequestration and degradation of PIF3 by DELLAs contribute to a reduction in PIF3 binding to its target genes. Thus, we show that promotion of PIF degradation by DELLAs is required to coordinate light and GA signals, and the dual regulation of transcription factors by DELLAs by both sequestration and degradation may be a general mechanism.

## Abstract (web summary)

Gibberellins (GA) negatively regulate light-mediated suppression of hypocotyl elongation in plants. Here, Li *et al*. show that GA-mediated destabilization of DELLA proteins promotes accumulation of the light-regulated PIF transcription factors thus contributing to the crosstalk between light and GA signalling.

## Graphical abstract

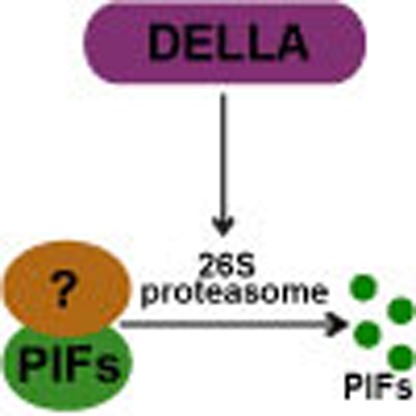

Light promotes plant photomorphogenesis, giving rise to open and expanded cotyledons, and short hypocotyls in light-grown *Arabidopsis* seedlings. In the dark, seedlings undergo skotomorphogenesis/etiolation, as characterized by closed cotyledons and elongated hypocotyls^1^. A subset of basic helix-loop-helix (bHLH) transcription factors, known as phytochrome-interacting factors (PIFs), has been reported to have a key role in etiolation and light-regulated plant development. PIF3 was the first characterized member of this gene family, identified by yeast two-hybrid screen using phytochrome B as the bait^2^. Mutation of PIF3 resulted in short hypocotyls in red light, indicating that PIF3 is a negative regulator of light signal transduction^3^,^4^. Several other homologous PIF proteins, including PIF1, PIF4, and PIF5, have also been reported to regulate photomorphogenesis^5^,^6^,^7^. A quadruple *pif* mutant (lacking PIF1, PIF3, PIF4, and PIF5) exhibits a striking constitutively photomorphogenic phenotype in the dark, which indicates that these four PIF factors act redundantly to promote etiolated growth^8^,^9^,^10^. In the dark, PIF proteins accumulate and directly regulate thousands of genes to maintain skotomorphogenesis^11^,^12^,^13^. Upon illumination, the photoactived phytochromes trigger PIFs' rapid phosphorylation and subsequently proteasome-mediated degradation^14^,^15^,^16^,^17^, leading to a cascade of transcriptional changes to promote photomorphogenesis^12^,^18^,^19^,^20^.

During seedling development, gibberellins (GAs) promote etiolated growth, thus showing the opposite effect to light on photomorphogenic programme^21^. Deficiency of GA (as in *ga1-3* mutant) induces partial constitutive photomorphogenesis phenotype in darkness, resulting in a loss of apical hook, open cotyledons and shortened hypocotyls^21^. DELLA proteins are the key repressors of almost all GA responses^22^. There are five DELLA proteins in *Arabidopsis* GA INSENSITIVE (GAI), REPRESSOR OF *ga1-3* (RGA), RGA-LIKE 1 (RGL1), RGL2 and RGL3, which have both distinct and overlapping functions^22^,^23^,^24^,^25^,^26^,^27^,^28^,^29^,^30^. In darkness, constitutively photomorphogenic phenotypes of *ga1-3* mutants can be almost fully suppressed by *rga* and *gai* null alleles, indicating that RGA and GAI are the two main DELLA members involved in GA-dependent repression of photomorphogenic growth in seedlings^21^,^31^,^32^. When GA is present, GA receptor GID1 binds to the DELLAs to form GID1–GA–DELLA complex, which triggers the ubiquitination and subsequent degradation of DELLA proteins by the 26S proteasome^33^,^34^,^35^. DELLAs have a conserved DELLA domain at the N terminus that is essential for GA-triggered protein degradation^22^. Deletion of the DELLA domain results in stabilization of these proteins and leads to a GA-unresponsive dwarf phenotype^22^.

How light and GAs antagonistically regulate hypocotyl elongation has been extensively analyzed in the past two decades. Two independent studies demonstrated that DELLAs can physically interact with and block PIF3 and PIF4 activities by sequestering the transcription factors from binding to their targets, which ultimately results in inhibition of hypocotyl elongation^36^,^37^. Further study showed that DELLAs also interact with the bHLH proteins PIF1 (also known as PIL5), PIF6 (also known as PIL2) and SPATULA(SPT)^38^. The sequestration of PIFs by DELLAs provided an important molecular link between light and GA signalling in regulating photomorphogenesis. Later, the model of sequestration was demonstrated to be a general mechanism for DELLAs to regulate transcription factors involved in other signalling pathways^39^,^40^,^41^,^42^,^43^,^44^. Beside the well-established sequestration action model, we are interested in exploring whether there are additional regulatory actions between DELLAs and PIFs in the crosstalk of light and GA signals.

We were intrigued by the observation that RGA and GAI proteins oscillate in a diurnal manner, with higher level during daytime and lower level at night, which is critical for the rhythmic growth of hypocotyls^45^. In contrast, as the prominent regulators of seedlings growth under the diurnal condition, PIF3 abundance accumulates in the dark to induce hypocotyls growth and it decreases after light illumination^46^. Following the clue of opposite oscillation patterns of DELLAs and PIF3 proteins, we focused on protein turnover as a potential regulatory mechanism. In this study, we report that DELLAs promote the degradation of PIF proteins through the ubiquitin–proteasome system. Thus, protein degradation is an additional mechanism by which DELLAs inactivate PIFs to coordinate light and GA signals during plant development.

## Results

### DELLAs negatively regulate PIF3 protein abundance

**Figure 1:**
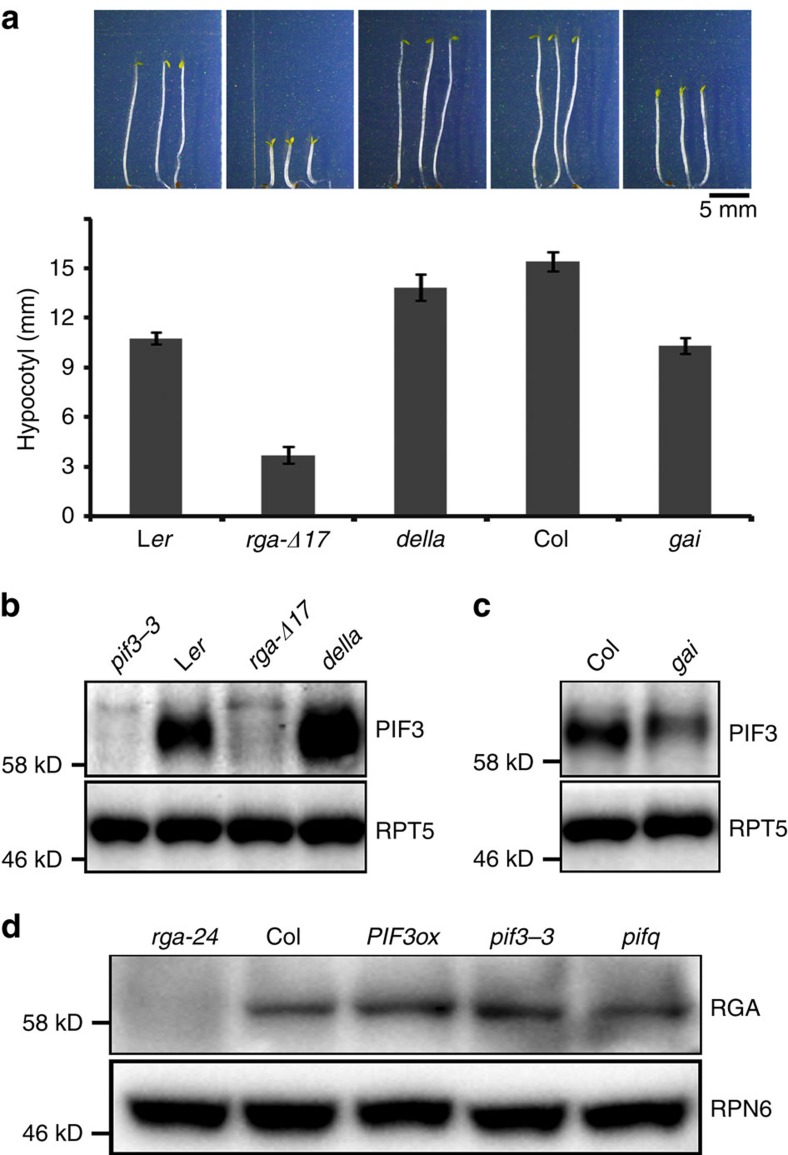
DELLAs negatively regulate PIF3 protein abundance in the dark. (**a**) Phenotypes and hypocotyl lengths of 4-day-old dark-grown *rga-Δ17*, *della*, *gai* and their wild-type counterparts. Means±s.d. were obtained from at least 30 seedlings. (**b**,**c**) Endogenous PIF3 protein levels in various DELLA mutants. Total proteins extracted from 4-day-old dark-grown seedlings were immunoblotted with anti-PIF3 and anti-RPT5 antibodies. RPT5 was used as a loading control. (**d**) Effects of PIFs on RGA protein levels. Four-day-old dark-grown seedlings were collected for protein extraction, and total proteins were immunoblotted with anti-RGA and anti-RPN6 antibodies. *PIF3ox* indicates *35S:PIF3-Myc*. RPN6 was used as a loading control. Each immunoblot result is the representative of at least three repeats.

Intrigued by opposite protein accumulation dynamics of DELLAs and PIF3 in a diurnal cycle^45^,^46^, we designed the experiments to ask whether DELLAs and PIF3 affect the protein level of each other. Two DELLA proteins RGA and GAI are the two main repressors of GA-mediated suppression of photomorphogenesis in darkness^21^, were used for the study. The GA-insensitive mutants *rga-Δ17* and *gai*, lacking 17 amino acids within the DELLA domain that is required for GA-induced degradation^23^,^24^,^26^, were used to examine PIF3 protein level. As shown before^26^, the *rga-Δ17* mutant exhibited shorter hypocotyls compared to its wild-type counterpart, L*er* in darkness (Fig. 1a). Accompanying this phenotype, endogenous PIF3 protein levels were dramatically reduced (Fig. 1b). Similarly, the non-degradable GAI mutant showed shortened hypocotyls and decreased PIF3 protein abundance in darkness (Fig. 1a,c). On the basis of the observed effects on hypocotyl length and PIF3 protein abundance, it seems that RGA has a more dominant role than GAI in inhibiting skotomorphogenesis. By contrast, *della* pentuple mutant exhibited elongated hypocotyl length and slightly increased PIF3 protein abundance compared with the wild type (Fig. 1a,b), further supporting a negative role of DELLAs in regulating skotomorphogenesis and PIF3. RGA and GAI had little effect on PIF3 transcript level (Supplementary Fig. 1a,b), suggesting that DELLAs negatively regulate PIF3 abundance mainly at the post-transcriptional level. To test whether PIF3 affects the abundance of RGA, we examined RGA protein levels in wild type, *35:PIF3-Myc*, *pif3-3* and *pifq* (*pif1 pif3 pif4 pif5*) seedlings, and the results suggested that PIF3 has no obvious effect on RGA protein level (Fig. 1d).

### Induction of DELLA proteins triggers PIF3 degradation

**Figure 2:**
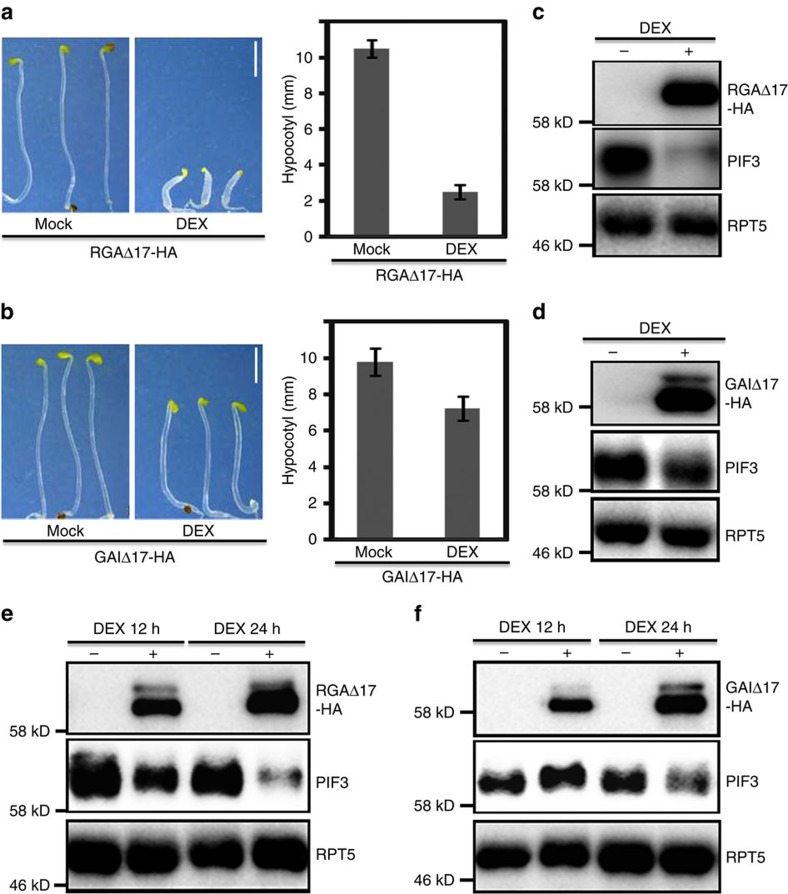
Induction of DELLAs expression leads to decline of PIF3 protein levels in the dark. (**a**,**b**) Phenotypes and hypocotyl lengths of 4-day-old RGAΔ17-HA and GAIΔ17-HA seedlings treated without or with 1 μM DEX in the dark. Means±s.d. were calculated from at least 30 seedlings. All images were under the same magnification. Scale bar, 2 mm. (**c**,**d**) RGAΔ17-HA, GAIΔ17-HA and PIF3 protein levels in the plants as shown in (**a**) and (**b**). Total proteins were analyzed by immunoblots using anti-HA, anti-PIF3 and anti-RPT5. (**e**,**f**) Analysis of PIF3 protein levels after transient induction of RGAΔ17-HA or GAIΔ17-HA. Four-day-old dark-grown RGAΔ17-HA or GAIΔ17-HA seedlings were collected and infiltrated without or with 10 μM DEX for 12 h or 24 h. Total proteins were analyzed by immunoblots using anti-HA, anti-PIF3 and anti-RPT5. Each immunoblot result is the representative of at least three repeats, and RPT5 was used as a loading control.

To further investigate the role of DELLAs in regulating PIF3 protein abundance, we generated transgenic *Arabidopsis* plants harbouring the HA-tagged RGAΔ17 or GAIΔ17 under the control of a dexamethasone (DEX)-inducible promoter. RGAΔ17-HA and GAIΔ17-HA seedlings without treatment showed typical etiolated phenotypes in darkness, while DEX-treated seedlings displayed retarded hypocotyl growth (Fig. 2a,b), similar to *rga-Δ17* or *gai* mutants (Fig. 1a). Immunoblot analysis showed that both RGAΔ17-HA and GAIΔ17-HA proteins accumulated to high levels after DEX induction (Fig. 2c,d). These results demonstrated that DEX-induced RGAΔ17-HA and GAIΔ17-HA fusion proteins were biologically functional, as they were able to shortcut endogenous GA action and induce a photomorphogenic-like phenotype. The amount of PIF3 was markedly decreased when RGAΔ17-HA or GAIΔ17-HA accumulated in the presence of DEX (Fig. 2c,d), which is consistent with the results in Fig. 1. We next performed transient induction of the transgenes. After 12 or 24 h DEX treatment, as RGAΔ17-HA protein accumulated, PIF3 protein abundance dramatically reduced (Fig. 2e). Similarly, the accumulation of GAIΔ17-HA after 24 h DEX treatment also led to the reduction of PIF3 protein (Fig. 2f). There were no obvious changes of PIF3 transcripts during the DEX induction (Supplementary Fig. 1c,d), indicating that DELLAs negatively regulated PIF3 mainly at the protein level.

### DELLA control of PIF levels correlates with hypocotyl length

**Figure 3:**
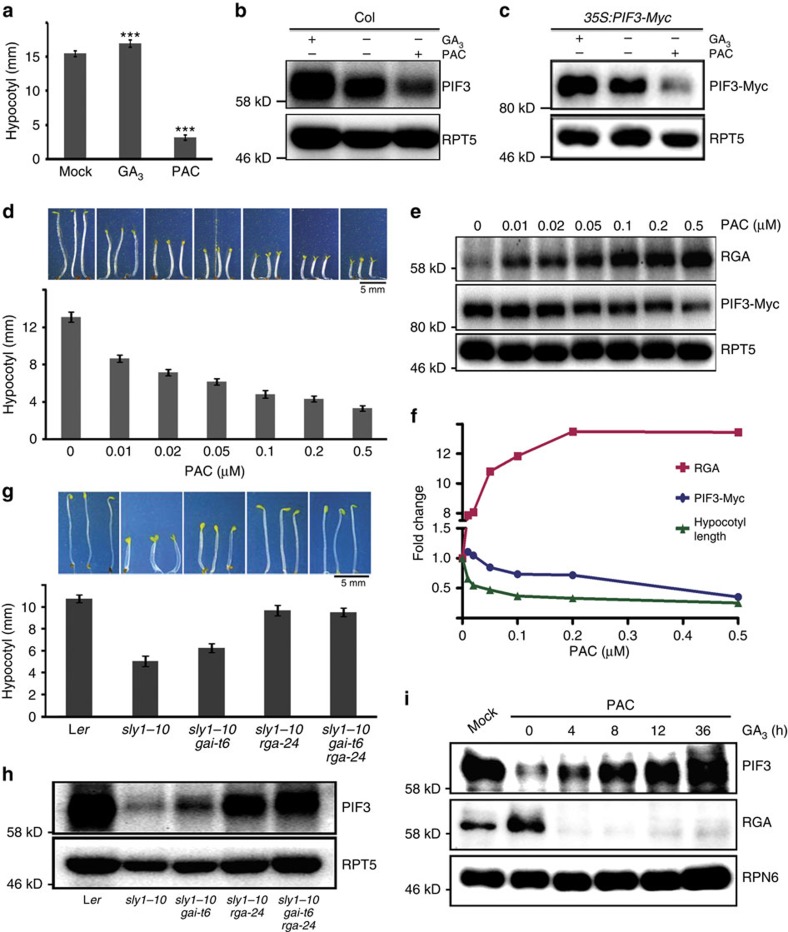
Regulation of PIF3 protein abundance by DELLAs correlates with GA-regulated hypocotyl elongation. (**a**) Hypocotyl lengths of 4-day-old dark-grown wild-type (Col) seedlings treated with GA_3_ (10 μM) or PAC (0.5 μM). Statistical significance was determined using the Student's *t* tests between the GA_3_ or PAC treatment and Mock. ***P<0.001. (**b**,**c**) Effects of GA_3_ or PAC treatment on PIF3 and PIF3-Myc protein abundance. The seedlings were grown in the medium with indicated supplements, and total proteins were analyzed by immunoblots using anti-PIF3, anti-Myc and anti-RPT5. (**d**) Phenotypes and hypocotyl lengths of 4-day-old dark-grown *35S:PIF3-Myc* seedlings treated with different concentrations (0, 0.01, 0.02, 0.05, 0.1, 0.2 and 0.5 μM) of PAC. All images were under the same magnification. (**e**) RGA and PIF3-Myc protein levels of the seedlings as shown in (**d**). (**f**) Relative changes of RGA, PIF3-Myc, and hypocotyl lengths of the seedlings shown in (**d**,**e**). The values of starting points were set to 1. (**g**) Phenotypes and hypocotyl lengths of 4-day-old dark-grown seedlings. All images were taken under the same magnification. (**h**) Endogenous PIF3 protein levels of the seedlings shown in (**g**). (**i**) PIF3 protein levels in Col seedlings treated with PAC and GA. Seedlings were grown on 0.2 μM PAC-containing medium in darkness for 4 days and then treated with 100 μM GA_3_ for indicated times. Each immunoblot result is the representative of at least three repeats, and RPN6 was used as a loading control. In (**a**), (**d**) and (**g**), Means±s.d. were obtained from at least 20 seedlings.

As the central repressors in the GA signalling pathway, abundance of DELLAs is tightly controlled by GA^33^,^34^,^35^. Since DELLAs were shown to regulate PIF3 protein abundance (Figs 1 and 2) and PIF3 promotes hypocotyls elongation^3^,^46^, we speculated that the abundance of PIFs might be regulated by GA content, which may further mediate hypocotyl elongation. As expected, GA_3_ (an active form of GA) treatment promoted, whereas paclobutrazol (PAC; a GA biosynthesis inhibitor) treatment inhibited hypocotyl elongation of wild-type (Col) plants (Fig. 3a and Supplementary Table 1). As shown in Fig. 3b, GA_3_ treatment promoted endogenous PIF3 protein accumulation, while PAC treatment caused an obvious decrease of PIF3 abundance. Consistently, GA_3_ or PAC treatment of seedlings of two other wild-type *Arabidopsis* ecotypes, Wassilewskija (Ws) and Landsberg *erecta* (L*er*), showed similar results (Supplementary Fig. 2). To exclude the possibility of regulation at the transcript level, *35S:PIF3-Myc* seedlings were also treated with GA_3_ or PAC. Similar to the results in wild type (Fig. 3b), GA_3_ increased, while PAC repressed, PIF3-Myc protein abundance (Fig. 3c).

To quantitatively assess the relationships between DELLAs, PIF3 and hypocotyl elongation, we examined RGA and PIF3 protein levels, and hypocotyl length of *35S:PIF3-Myc* seedlings grown in the presence of various PAC concentrations. With increasing dose of PAC, hypocotyl elongation of dark-grown seedlings declined progressively (Fig. 3d). In these seedlings, RGA accumulated, while PIF3-Myc decreased gradually (Fig. 3e). By calculating the relative change of hypocotyl length and protein abundance of RGA and PIF3-Myc, we found that the attenuating effect of RGA on PIF3 abundance correlated well with hypocotyl growth (Fig. 3f).

To further analyze the significance of DELLA's negative regulation on PIF3 abundance in the GA signalling pathway, we checked the phenotypes and PIF3 protein abundance in dark-grown *sly1-10*, *sly1-10 gai-t6*, *sly1-10 rga-24* and *sly1-10 gai-t6 rga-24* seedlings (Fig. 3g,h). SLEEPY1 (SLY1) is an E3 ligase component responsible for the ubiquitination of DELLA proteins, and its mutant (*sly1-10*) contains elevated levels of RGA and GAI^33^,^34^,^35^. The constitutive photomorphogenic phenotype of *sly1-10* could be partially suppressed by the absence of GAI (*gai-t6*) and RGA (*rga-24*) (Fig. 3g), which indicates that accumulation of GAI and RGA can account for the short hypocotyl phenotype of *sly1-10*. PIF3 protein abundance was then examined in these mutants and the extremely low level of PIF3 in *sly1-10* was partially rescued by the absence of RGA and GAI (Fig. 3h). This is consistent with the notion that over-accumulation of RGA and GAI proteins in *sly1-10* promote degradation of PIF3. For the regulation of PIF3 protein level and hypocotyl elongation, the effect of RGA is stronger than GAI (Fig. 3g,h), similar to the results in Figs 1 and 2. These data suggest that the reduction of PIF3 abundance by DELLAs may have a key role in suppressing GA-mediated hypocotyl elongation.

DEX-induced RGA accumulation led to the PIF3 degradation within 12 h (Fig. 2). To determine how fast DELLAs' regulation of PIF3 responds to the change of GA levels, we applied GA_3_ to seedlings grown on medium containing PAC and checked how PIF3 level recovered. In the plants with PAC treatment, RGA protein accumulated and PIF3 protein decreased. GA_3_ application led to a quick reduction of RGA protein levels, while PIF3 abundance increased within hours and almost fully recovered by 12 h of GA_3_ treatment (Fig. 3i). Together, the data in Figs 2 and 3 suggest that DELLA-mediated degradation may provide the plant with a tunable way of regulating the abundance of PIFs in response to a variety of cues that modulate GA metabolism.

### DELLAs contribute to diurnal oscillation of PIF3 levels

**Figure 4:**
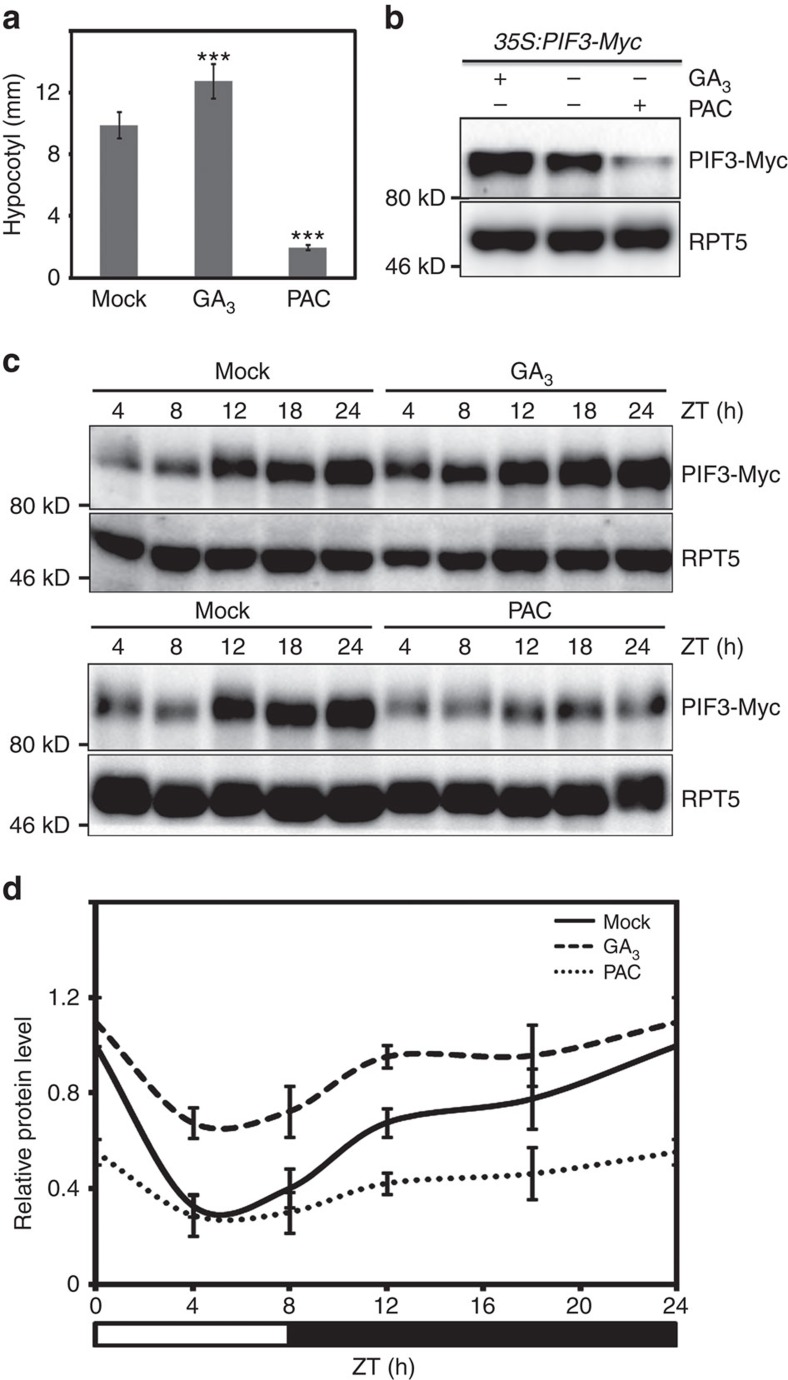
DELLAs negatively regulate PIF3 protein abundance under continuous light and diurnal conditions. (**a**) Hypocotyl lengths of 4-day-old *35S:PIF3-Myc* seedlings treated with GA_3_ (10 μM) or PAC (0.5 μM) under continuous red light. Means±s.d. were calculated from at least 30 seedlings. Statistical significance was determined using the Student's *t* tests between the GA_3_ or PAC treatment and Mock. ***P<0.001. (**b**) PIF3-Myc protein levels of the seedlings shown in (**a**). Total proteins were analyzed by immunoblots using anti-Myc and anti-RPT5. RPT5 was used as a loading control. (**c**) Levels of PIF3-Myc proteins in seedlings grown under diurnal conditions. *35S:PIF3-Myc* seedlings were grown on the medium without treatment, with GA_3_ (10 μM), or with PAC (0.5 μM) under short-day condition (8 h white light/16 h darkness) for 5 days, and samples were collected at the indicated times. (**d**) The quantification of the western blot results from (**c**) using image J software. PIF3-Myc protein levels were normalized to RPT5 loading control. The value of Mock at ZT24 was set to be 1. Quantitative data are shown as means±s.e.m., *n*=3.

To investigate whether DELLAs also promote PIF3 degradation under light conditions, *35S:PIF3-Myc* seedlings were grown on medium containing GA_3_ or PAC under continuous red light and PIF3-Myc protein levels were checked. As shown in Fig. 4a,b, DELLAs negatively regulate hypocotyl elongation and PIF3 protein abundance under red light. Considering that DELLAs and PIF3 show opposite protein abundance dynamics in a diurnal cycle^45^,^46^, we were interested in whether DELLAs help forming the oscillatory wave of PIF3 levels under diurnal conditions. To test this, we examined the pattern of PIF3-Myc protein levels in *35S:PIF3-Myc* seedlings treated with or without GA_3_ or PAC under diurnal short-day conditions. As shown in Fig. 4c,d, PIF3-Myc protein abundance was low in daytime and accumulated to higher levels in the night, which was consistent with previous studies^46^. On GA_3_ application, PIF3-Myc increased throughout the day compared to mock treatment, and the increase was larger in daytime and early night (at ZT4, 8, 12 h) than in mid- and late night (at ZT18, 24 h) (Fig. 4c,d), which weakened the oscillation patterns of PIF3 protein. These results suggested that DELLAs contribute to the oscillation patterns of PIF3 protein levels in plants grown in diurnal light/dark conditions. On PAC treatment, a condition that increased DELLA abundance, not only did PIF3-Myc levels decrease sharply, but also its oscillation pattern was dramatically impaired (Fig. 4c,d). These results further support the contribution of DELLAs to PIF3 oscillation under diurnal conditions.

### DELLAs and phyB/LRBs mediate PIF degradation independently

**Figure 5:**
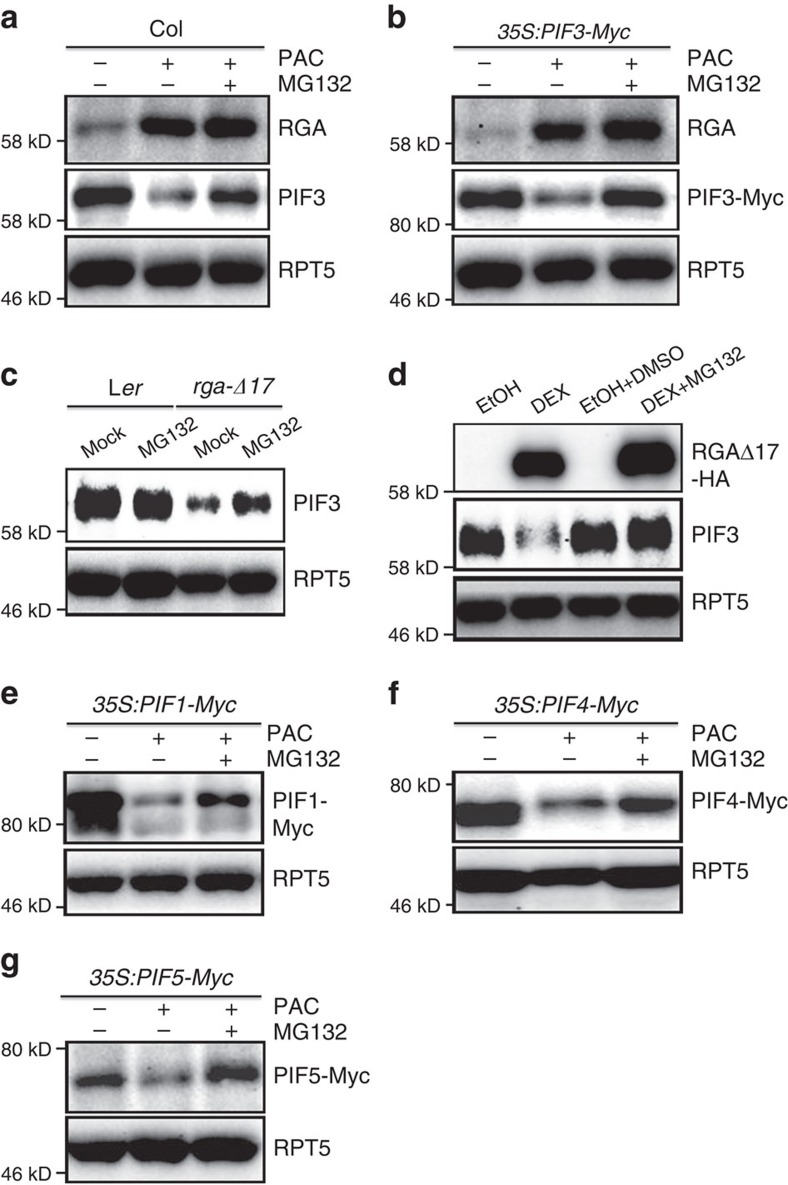
DELLAs promote the degradation of PIF proteins via the ubiquitin–proteasome system. (**a**,**b**) Effects of PAC (0.5 μM) and MG132 (100 μM) treatments on RGA, PIF3 and PIF3-Myc protein levels in 4-day-old dark-grown seedlings. (**c**) Effects of MG132 on PIF3 protein levels in 4-day-old dark-grown *rga-Δ17* seedlings. (**d**) Effects of MG132 on PIF3 protein levels after transient induction of RGAΔ17-HA. Four-day-old dark-grown RGAΔ17-HA seedlings were treated with DEX or DEX plus MG132 for 24 h. EtOH and DMSO are the solvents for DEX and MG132, respectively. (**e–g**) Effects of combinational PAC and MG132 treatments on PIF1-Myc, PIF4-Myc and PIF5-Myc protein levels in 4-day-old dark-grown seedlings. Each immunoblot result is the representative of at least three repeats, and RPT5 was used as a loading control.

We next tested whether DELLAs modulate the abundance of PIF3 via the ubiquitin–proteasome system. We found that PAC-induced reduction of endogenous PIF3 levels in wild-type seedlings or seedlings ectopically expressing the PIF3-Myc protein was inhibited by treatment with MG132, a 26S proteasome inhibitor (Fig. 5a,b). Similarly, MG132 treatment also increased PIF3 protein levels in *rga-Δ17* mutant seedlings in the dark (Fig. 5c). Moreover, MG132 completely blocked PIF3 degradation in RGAΔ17-HA seedlings with DEX treatment (Fig. 5d). Taken together, these results showed that inhibiting proteasome activity is able to abolish the degradation of PIF3, which would otherwise take place under circumstances where DELLA proteins accumulate, and thus suggests that DELLAs promote PIF3 protein degradation through the ubiquitin–proteasome pathway.

Since PIF3 functions redundantly with its three homologues, PIF1, PIF4 and PIF5, to repress photomorphogenesis in darkness^8^,^9^,^10^, and DELLAs sequester and inhibit transcriptional activity of both PIF3 and PIF4 (refs 36, 37), we tested whether DELLAs also negatively regulate protein abundance of PIF1, PIF4 and PIF5. As shown in Fig. 5e–g, the abundance of all three PIF-Myc proteins was reduced in the *35S:PIF1-Myc*, *35S:PIF4-Myc* and *35S:PIF5-Myc* seedlings after PAC treatment. Furthermore, PAC triggered reduction of PIF-Myc abundance could be inhibited by MG132 treatment. Collectively, these data indicate that DELLAs promote the degradation of these four PIF proteins via the proteasome.

**Figure 6 f6:**
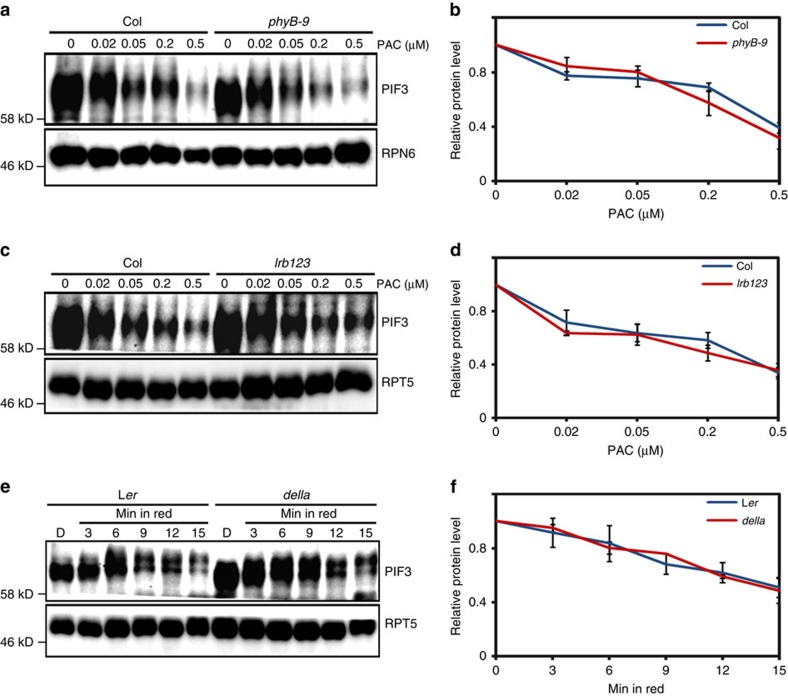
DELLAs and phyB/LRBs regulate the degradation of PIF3 independently. (**a**) Endogenous PIF3 protein levels in the 4-day-old dark-grown seedlings of *phyB-9* mutant and its wild-type counterparts grown on medium containing different concentrations (0, 0.02, 0.05, 0.2 and 0.5 μM) of PAC. RPN6 was used as a loading control. (**b**) The quantification of the western blot results from (**a**) using image J software. PIF3 protein levels were normalized to RPN6 loading control. The value of starting point was set to 1. Quantitative data are shown as means±s.e.m., *n*=3. (**c**) Endogenous PIF3 protein levels in 4-day-old dark-grown *lrb1lrb2lrb3* triple-mutant (*lrb123*) and wild-type seedlings grown on medium containing different concentrations (0, 0.02, 0.05, 0.2 and 0.5 μM) of PAC. RPT5 was used as a loading control. (**d**) The quantification of the western blot results from (**c**) using image J software. PIF3 protein levels were normalized to RPT5 loading control. The value of starting point was set to 1. Quantitative data are shown as means±s.e.m., *n*=3. (**e**) Endogenous PIF3 protein levels in 4-day-old seedlings of *della* mutant and its wild-type counterparts during dark to red light transition. Dark-grown seedlings were irradiated with red light (5 μmol m^−2 ^s^−1^) for the indicated times. RPT5 was used as loading control. (**f**) The quantification of the western blot results from (**e**) using image J software. PIF3 protein levels were normalized to RPT5 loading control. The value of starting point was set to 1. Quantitative data are shown as means±s.e.m., *n*=3.

The light-activated photoreceptor phyB interacts with PIF3 and recruits LRB1/2/3 containing E3 ligases to ubiquitinate PIF3 (ref. 16). To address whether phyB and LRBs are involved in DELLA-mediated PIF degradation, we treated *phyB-9* and *lrb123* mutants with different concentrations of PAC (Fig. 6a–d and Supplementary Fig. 3). The results showed that PAC can induce PIF3 destruction in *phyB-9* and *lrb123* mutants as effectively as in wild type. These results indicate that phyB and LRBs are not required for DELLA-mediated PIF3 degradation. Conversely, we investigated whether the *della* pentuple mutant affect light-triggered degradation of PIF3 protein. As shown in the Fig. 6e,f, the red light-induced degradation rate of PIF3 in *della* mutant is similar to that in wild type, indicating that DELLAs are not required for red light-induced PIF3 degradation via phyB/LRBs. Thus, DELLAs and phyB/LRBs represent two independent pathways that mediate degradation of PIF3.

COP1 and DET1 proteins have been shown to maintain PIF3 protein stability, and PIF3 protein can hardly be detected in *cop1-4* or *det1-1* mutant^47^,^48^. Our analyses showed that GA and PAC could not modulate PIF3 abundance without the presence of COP1 or DET1, indicating that COP1 and DET1 are essential for GA-mediated PIF3 accumulation (Supplementary Fig. 4).

### DELLAs regulate PIF3 by both sequestration and degradation

Previous reports indicated that DELLAs interact with PIF3 and prevent PIF3 from binding to its target genes^37^. In this study, we found that DELLAs can promote the degradation of PIF3. To dissect the specific contributions of these two forms of regulation, we examined the promoter occupancy of PIF3-Myc by Chromatin ImmunoPrecipitation (ChIP) experiments, as the readout of PIF3 activity under different PAC and/or MG132 treatments.

**Figure 7:**
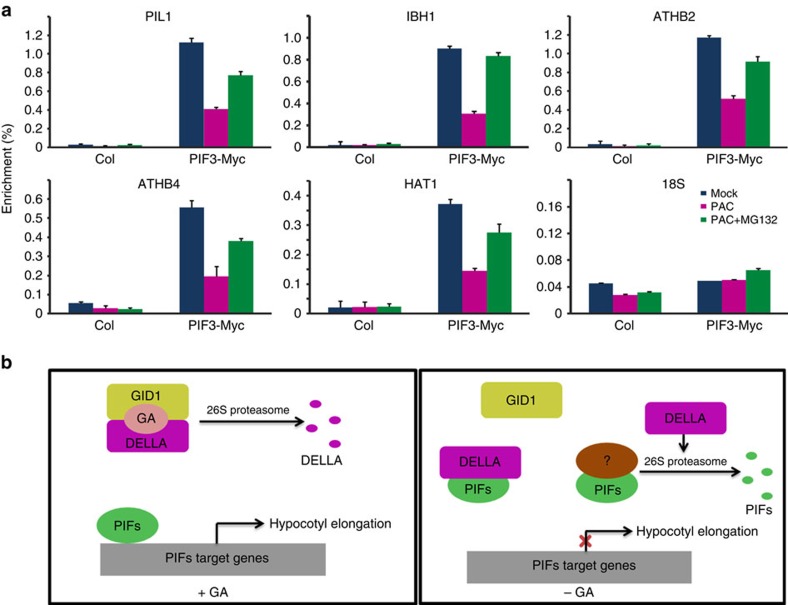
Both sequestration and degradation of PIF3 by DELLAs reduced the binding activity of PIF3 to its target genes. (**a**) The ChIP-qPCR analysis of PIF3-Myc binding to its target genes. The 4-day-old wild-type (Col) and *35S:PIF3-Myc* seedlings grown in the dark on regular medium, or on medium with 0.5 μM PAC and then treated with DMSO or 100 μM MG132 for 4 h, were used for ChIP-qPCR analyses. A 18S rDNA was used as a non-binding control. The data was calculated from three biological replicates. RGA and PIF3-Myc protein levels in these seedlings are shown in Fig. 5b and Supplementary Fig. 5. (**b**) A model showing that DELLAs inhibit PIFs via both sequestration and degradation to regulate hypocotyl elongation. GA promotes the degradation of DELLAs via 26S proteasome system, and PIFs released from DELLAs can enhance hypocotyl elongation (left panel). When GA is absent, more DELLAs are accumulated, which inhibit the functions of PIFs at two levels: First, DELLAs sequestrate PIFs from binding to their targets^36^,^37^; Second, DELLAs promote the degradation of PIFs via 26S proteasome. These inhibitions on PIFs together reduce hypocotyl elongation (right panel).

Seedlings treated with PAC had increased RGA levels and reduced PIF3-Myc levels (Fig. 5b and Supplementary Fig. 5), presenting a situation in which presumably both sequestration and degradation mechanisms would have a role in regulating PIF3 activity. In contrast, treatment of seedlings with both PAC and MG132 increased RGA abundance, while PIF3-Myc levels were almost the same as mock-treated seedlings, presenting a situation in which only sequestration is likely to have a role. We then analyzed the binding of PIF3 to its known target genes (PIL1, IBH1, ATHB2, ATHB4 and HAT1)^13^. As shown in Fig. 7a, the promoters of these five genes were significantly enriched in the ChIP samples of untreated *35S:PIF3-Myc* seedlings (Mock). In PAC-treated seedlings, the enrichment of these promoters was significantly decreased, indicating that sequestration and degradation together strongly inhibit the binding of PIF3 to its target genes. On the other hand, in seedlings treated with both PAC and MG132, the enrichment levels, although still decreased when compared to mock, were clearly higher than those treated with PAC alone, indicating that sequestration by itself can already inhibit the binding of PIF3 to its targets, while degradation provides additional level of inhibition, presumably reducing PIF3 activity even further. Treatment of MG132 alone without PAC did not affect PIF3-Myc or RGA level (Supplementary Fig. 6a), nor the binding of PIF3-Myc on the targets (Supplementary Fig. 6b).

Although DELLA proteins have no classical DNA-binding domains, they have been shown to directly target some GA-responsive genes such as *SCL3* (refs 49, 50). To test whether DELLAs potentially interfere in PIF3 binding to its target genes, we conducted a RGA ChIP analysis using the inducible HA-tagged RGAΔ17 transgenic plants. Our data indicated that DEX-inducible RGAΔ17-HA protein bound strongly to the *SCL3* promoter, but not to the promoters of PIF3 target genes (Supplementary Fig. 7). These results suggest that DELLA proteins would not bind PIF3 target genes.

Taken together, these data suggest that DELLAs can inhibit PIF3 activity via a dual regulatory mechanism, which involves both sequestration and degradation.

## Discussion

Light and GA antagonistically control hypocotyl elongation of seedlings. In this crosstalk, DELLA proteins sequestrate PIF3 and PIF4, and repress their DNA-binding ability, which explains the antagonistic control of hypocotyl elongation by light and GA^36^,^37^. In this study, we found that DELLAs negatively regulate PIF3 protein abundance under both continuous dark and light conditions (Figs 1, 2, 3, 4). In addition to PIF3 and PIF4, DELLAs also interact with PIF1 and PIF5 (Supplementary Fig. 8 and ref. 38), and promote degradation of these PIFs via the ubiquitin–proteasome system (Fig. 5). The reduction of PIF3 abundance by DELLAs contributes significantly to the oscillatory patterns of PIF3 protein level in plants growing in diurnal light/dark conditions (Fig. 4c,d). Furthermore, we showed that both the sequestration and degradation contribute to the inhibition of PIF3 activity (Fig. 7a). Therefore, degradation is a new level of regulation of DELLAs on PIFs, in addition to the previously demonstrated regulation by sequestration. Together, we propose a model that illustrates how GA and light signals coordinate to regulate plant hypocotyl elongation (Fig. 7b). Briefly, we propose that when GA is present, it promotes the formation of the GA–GID1–DELLA complex and leads to the rapid ubiquitination and degradation of DELLAs, thus releasing PIFs to promote hypocotyl elongation. In contrast, when GA is absent, we propose that DELLA proteins accumulate and inhibit the function of PIFs by both sequestrating them from binding to their target promoters as well as by promoting their degradation via the ubiquitin–proteasome system, and thus hypocotyl elongation is inhibited. DELLA-induced PIF3 degradation is independent of the light-mediated PIF3 degradation pathway, as it can occur in the absence of activated phyB and LRBs E3 ligase system. As to how DELLAs mediate the PIF3 abundance, COP1 and DET1 proteins were shown to be essential for GA-mediated PIF3 accumulation (Supplementary Fig. 4), but the E3 ligase through which DELLAs trigger PIF3 degradation is still unknown. GA_3_ application led to a quick reduction of RGA protein levels, but PIF3 abundance was almost fully recovered after 12 h treatment (Fig. 3i). During this period, the unknown E3 ligase responsible for DELLAs-mediated PIF3 degradation may be de-activated, and then PIF3 proteins may accumulate gradually.

GAs can also coordinate with other signals to regulate plant development. For example, core transcription factors in several different signalling pathways, including BZR1, EIN3, JAZ and WD-Repeat/bHLH/MYB complex, were shown to be sequestrated and inhibited by DELLAs, similar to PIF3 and PIF4 (refs 36, 37, 38, 39, 40, 41, 42, 43, 44). These results suggest that sequestration is a general mechanism by which DELLAs inhibit transcription factors. Among these transcription factors, BZR1 was the only one for which DELLA was reported to promote a reduction in protein levels^41^. Here, we showed that DELLAs can promote the degradation of PIF1, PIF3, PIF4 and PIF5. Extending from this finding, we propose that controlling protein abundance of transcription factors represents another general mechanism of how DELLAs may regulate their targets. To test this hypothesis, we examined EIN3 from the ethylene signalling pathway. A previous study showed that DELLAs sequester EIN3, but GA_3_ did not markedly affect EIN3 protein abundance^42^. Consistently, GA treatments did not noticeably affect the abundance of EIN3-GFP and EIN3-FLAG proteins in our experiment; however, PAC treatments dramatically reduced their abundance (Supplementary Fig. 9a,b). It is possible that EIN3 accumulates to a high level in darkness, and is not sensitive to exogenous GA, which decreases DELLA levels but could not further increase EIN3 abundance. On the other hand, highly elevated levels of DELLAs after PAC treatment may promote EIN3 degradation. Taken together, our data suggests that the inhibition of transcription factors by DELLAs is mediated through both sequestration and degradation.

## Methods

### Plant materials and growth conditions

The wild-type *Arabidopsis* ecotypes used in this study were Columbia-0 (Col-0), Landsberg *erecta* (L*er*) and Wassilewskija (WS). The mutants and transgenic lines were described previously: *pif3-3* (ref. 4), *35S: PIF1-Myc, 35S: PIF3-Myc, 35S: PIF4-Myc* and *35S: PIF5-Myc* (ref. 48), *rga-24* (ref. 25)*, rga-Δ17* (ref. 26), *della* (*gai-t6 rga-t2 rgl1-1 rgl2-1 rgl3-1*) (ref. 37), *sly1-10 gai-t6*, *sly1-10 rga-24*, *sly1-10 gai-t6 rga-24* (ref. 34), *phyB-9* (ref. 51), *lrb123* (ref. 16), *cop1-4* (ref. 52), *det1-1*(ref. 53), *35S: EIN3-GFP*/*ein3 eil1* (ref. 54) and *35S: EIN3-FLAG*/*ein3 eil1* (ref. 55). Since homozygous *rga*-*Δ17* is sterile^26^, the homozygous *rga*-*Δ17* seedlings segregated from the progenies of heterozygous *rga*-*Δ17* were used for experiments. *gai* (Col background) is a gift from Xiangdong Fu of IGDB. All seeds were surface-sterilized and sown on Murashige and Skoog (MS) medium containing 1% sugar. Seeds were cold-treated at 4 °C for 4 days in the dark before germination. The seedlings were grown in the dark, under continuous red light (0.5 μmol m^−2 ^s^−1^) or under short-day conditions (8 h white light (85 μmol m^−2 ^s^−1^) +16 h dark) for the indicated times, unless indicated otherwise. Manipulation of seedlings in darkness was performed under dim green light.

### Generation of transgenic plants

DNA fragments containing two deletion mutants (*RGAΔ17* and *GAIΔ17*) without stop codons were amplified and inserted into the XmaI and PstI restriction sites of a pBSK-derived plasmid containing triple HA-tag to make constructs pBSK-RGAΔ17-HA and pBSK-GAIΔ17-HA. Further, the fragments *RGAΔ17-HA* and *GAIΔ17-HA* were amplified from pBSK-RGAΔ17-HA or pBSK-GAIΔ17-HA, and these fragments were digested with the SalI and SpeI, and then ligated into the XhoI and SpeI digested binary vector pTA7002 (ref. 56) to generate pTA7002-RGAΔ17-HA and pTA7002-GAIΔ17-HA constructs. These binary constructs were introduced into the GV3101 strain of *Agrobacterium* and transformed into *della* mutant plants, using the floral dip transformation method^57^. The transformants were selected on MS medium containing 50 mg ml^−1^ hygromycin B (Sigma-Aldrich), and named as RGAΔ17-HA and GAIΔ17-HA, respectively. The primers are listed in Supplementary Table 2.

### Plant treatments

GA_3_ (GA), paclobutrazol (PAC) and DEX were dissolved in ethanol. The proteasome inhibitor MG132 was dissolved in DMSO. For continuous GA_3_ and PAC treatments, after the seeds were induced by white light for germination on regular MS medium, they were transferred to medium containing GA_3_ (10 μM), PAC (0.5 μM) and ethanol (0.01% (v/v), as control), and then grown in the dark for 4 days. For the transient treatment by GA_3_, the seedlings were collected and vacuum-infiltrated with liquid MS medium containing 100 μM GA_3_ or ethanol alone (as control) for 10 min and then kept immersed in the same solution for the indicated times. For continuous DEX treatment, *Arabidopsis* seedlings were grown on the MS medium containing 1 μM DEX or ethanol alone (as control). For the transient induction by DEX, the 4-day-old seedlings were vacuum-infiltrated with liquid MS medium containing 10 μM DEX or ethanol alone (as control) for 10 min and then kept immersed in the same solution for the indicated times. For MG132 treatment, the 4-day-old seedlings were vacuum-infiltrated with liquid MS medium containing 100 μM MG132 (dissolved in DMSO) or DMSO alone (as control) for 10 min and kept immersed in the same solution for 4 h unless indicated otherwise.

### Protein extraction and Immunoblots

Total proteins were extracted by homogenizing seedlings using denaturing buffer (100 mM NaH_2_PO4, 10 mM Tris–HCl pH 8.0, 8 M urea, 1 mM phenylmethylsulfonyl fluoride, and × 1 complete protease inhibitor mixture (Roche). Seedlings were growing in the dark for 4 days unless specific indications. Extracts were centrifuged at 16,000*g* for 10 min at 4 °C, and protein concentration in the supernatants was quantified by the Bradford assay. Aliquots of denatured total protein were separated on 8% SDS–PAGE gels and transferred to PVDF membranes. Anti-PIF3 purified antibody at 1:500 (v/v) dilution (ref. 48), anti-Myc polyclonal antibody (Sigma-Aldrich, Cat. No: C3956) at 1:1,000 (v/v) dilution, anti-HA antibody (Sigma-Aldrich, Cat. No: H9658) at 1:1,000 (v/v) dilution, anti-RGA antibody (Agrisera, Cat. No: AS111630) at 1:1,000 (v/v) dilution, anti-FLAG (Sigma-Aldrich, Cat. No: F3165) at 1:1,000 (v/v) dilution, anti-GFP (Abmart, Cat. No: M20004) at 1:1,000 (v/v) dilution, anti-RPN6 polyclonal antibody (ref. 58) at 1:2,000 (v/v) dilution, and anti-PRT5 polyclonal antibody (ref. 58) at 1:2,000 (v/v) dilution were used as primary antibodies. Each experiment was repeated at least three times, and one representative result was shown. Quantification results of immunoblots in Figs 3f, 4d, 6b, 6d-6f, [Fig f6], [Fig f6] were quantified by Image J software. Supplementary Figures 10–22 are original full versions of the immunoblot images.

### RNA extraction and quantitative RT-PCR

Total RNA was extracted using the RNeasy plant mini kit (Qiagen). cDNA was synthesized by ReverTra Ace qPCR RT Master Mix (TOYOBO). The quantitative RT-PCR analysis was performed using SYBR Premix Ex Taq (Takara) in an ABI 7500 fast real-time instrument. Each experiment was repeated with three biological samples, and RT-PCR reactions were performed with three technical replicates for each sample. The primers are listed in Supplementary Table 2.

### Hypocotyl lengths measurement

After the indicated times of growth and treatment, at least 30 seedlings were laid on the agar plates, and digital pictures were taken. Then, the hypocotyl lengths were measured using Image J software.

### *In vitro* pull down assays

The constructs for expressing His-PIF1, His-PIF3, GST-PIF4 and GST-PIF5 were described previously^48^. Full-length RGA fragment was inserted into pMal-C2X vector to fuse with maltose-binding protein (MBP). All constructs were expressed in *E. coli* strain BL21 under the induction of 1 mM IPTG (isopropyl-β-D-thiogalactopyranoside). Two micrograms MBP or MBP–RGA proteins were mixed with 2 μg His- or GST-tagged proteins in 500 μl binding buffer (20 mM Tris–HCl, pH 7.5, 150 mM NaCl and 0.1% Nonidet P-40), and the mixture was rotated at 4 °C for 2 h. The amylase agarose beads were washed with binding buffer for three times and then added into the mixture. Then, the mixture was rotated at 4 °C for another 2 h. After being washed for five times with binding buffer, the MBP resin was boiled with protein loading buffer and analyzed by immunoblots. Anti-His (Sigma-Aldrich, Cat. No: H1029-.2ML) at 1:1,000 dilution, anti-GST (Sigma-Aldrich, Cat. No: G1160-.2ML) at 1:5,000 dilution and anti-MBP (New England Biolabs, Cat. No: E8032S) at 1:5,000 dilution were used for the western blots.

### Bimolecular fluorescence complementation assay

The full-length cDNA of RGA was amplified and inserted into the SpeI and BamHI sites of pSY736 (YFP^N^) vector, resulting in plasmid YFP^N^-RGA. YFP^C^-PIF1, YFP^C^-PIF3, YFP^C^-PIF4 and YFP^C^-PIF5 plasmids were described previously^48^. The plasmids were extracted and concentrated to 2 mg ml^−1^. The particle-mediated transformation using onion epidermal cells was performed^59^. After 24 h of incubation, YFP signal was detected using a Zeiss LSM 710 confocal microscope. The primers used for plasmid construction were listed in Supplementary Table 2.

### Chromatin immunoprecipitations assay

ChIP assays were performed as described previously^37^. *35S:PIF3-Myc* seedlings were grown for 4 days on the MS medium containing 0.5 μM PAC or EtOH in darkness, and were collected and treated with DMSO or 100 μM MG132 for 4 h. RGAΔ17-HA seedlings were grown for 4 days in the dark, and were collected and infiltrated with or without 10 μM DEX for 24 h. The samples (2 g) were treated with 15 ml of 1% formaldehyde under vacuum infiltration for 15 min, and then 1 ml 2 M glycine was added to stop crosslinking for 5 min. For the ChIP analysis used *35S: PIF3-Myc* seedlings, the solubilized chromatin was immunoprecipitated by 30 μl EZview Red Anti-c-Myc Affinity Gel (Sigma-Aldrich, Cat. No: E6654) at 4 °C for 5 h. For the ChIP analysis using RGAΔ17-HA seedlings, the solubilized chromatin was mixed with 10 μl anti-HA antibody (Sigma) and incubated at 4 °C for 1 h. Then, 40 μl Dynabeads Protein G (Life Technologies, Cat. No: 10003D) was added, and the sample was incubated overnight at 4 °C. The coimmunoprecipitated DNA was recovered and analyzed by quantitative PCR. All primers used in ChIP assays were listed in Supplementary Table 2.

### Data availability

The authors declare that all data supporting the findings of this study are available within the article and its Supplementary Information files or are available upon request from the corresponding authors.

## Additional information

**How to cite this article**: Li, K. *et al*. DELLA-mediated PIF degradation contributes to coordination of light and gibberellin signalling in *Arabidopsis*. *Nat. Commun.* 7:11868 doi: 10.1038/ncomms11868 (2016).

## Supplementary Material

Supplementary Information Supplementary Figures 1 - 22 and Supplementary Tables 1 and 2
