# Supplementary material for: DELLA-mediated PIF degradation contributes to coordination of light and gibberellin signalling in Arabidopsis
Source: Nat Commun. 2016 Jun 10;7:11868. doi: 10.1038/ncomms11868 (PMC4906400; doi:10.1038/ncomms11868)
Supplement: Supplementary Information — Supplementary Figures 1 - 22 and Supplementary Tables 1 and 2 [file ncomms11868-s1.pdf]

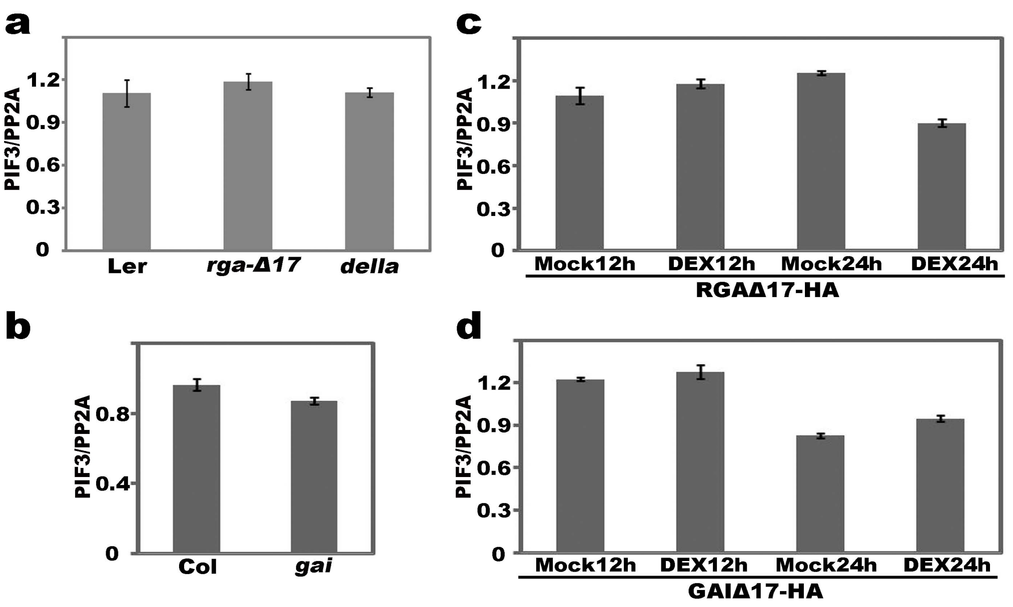

Supplementary Figure 1. Effects of DELLAs on PIF3 transcript levels. 4-day-old dark-grown seedlings under the indicated treatments were collected for RNA extraction and RT-PCR. PP2A served as an internal control. Quantitative data are shown as mean ± s.d. (n=3).

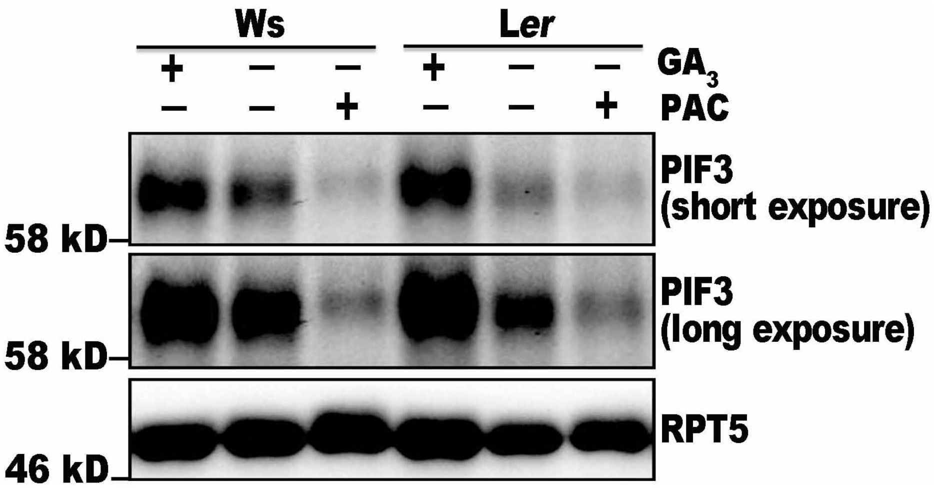

Supplementary Figure 2. GA positively regulates PIF3 protein abundance in different ecotypes of *Arabidopsis*. 4-day-old Ws and Ler seedlings were grown in the dark on medium with indicated supplements, and total proteins were analyzed by immunoblots using anti-PIF3 and anti-RPT5. RPT5 was used as a loading control.

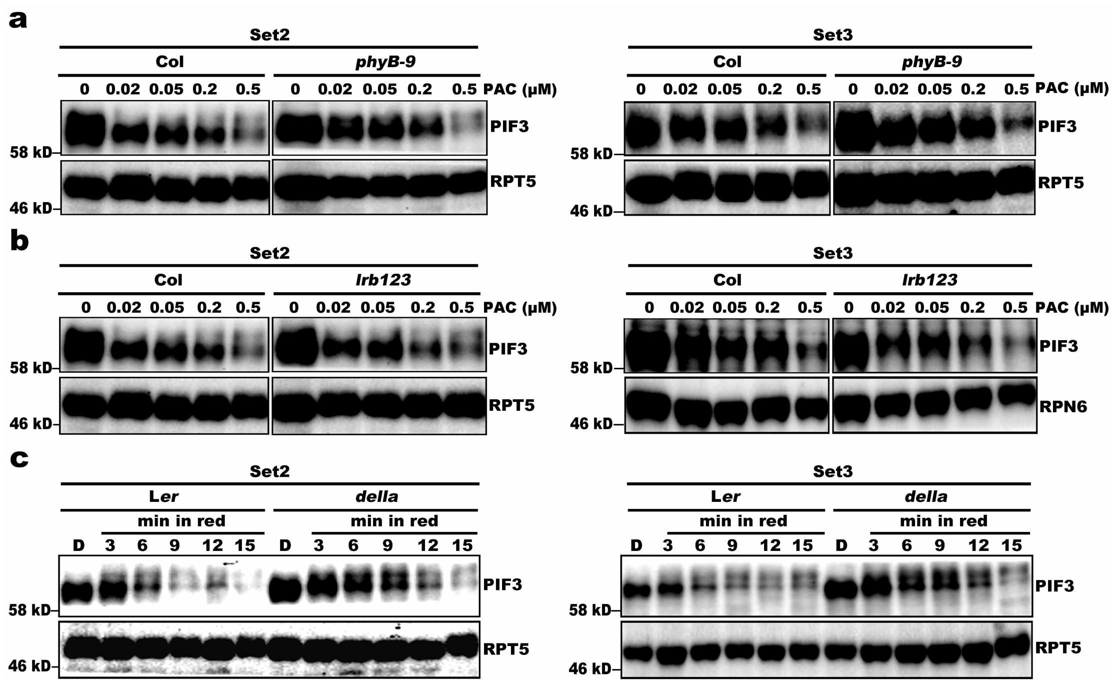

Supplementary Figure 3. Additional biological repetitions used for the quantificational analyses in Fig.6 b,d,f. The experimental conditions are identical to those in Fig. 6.

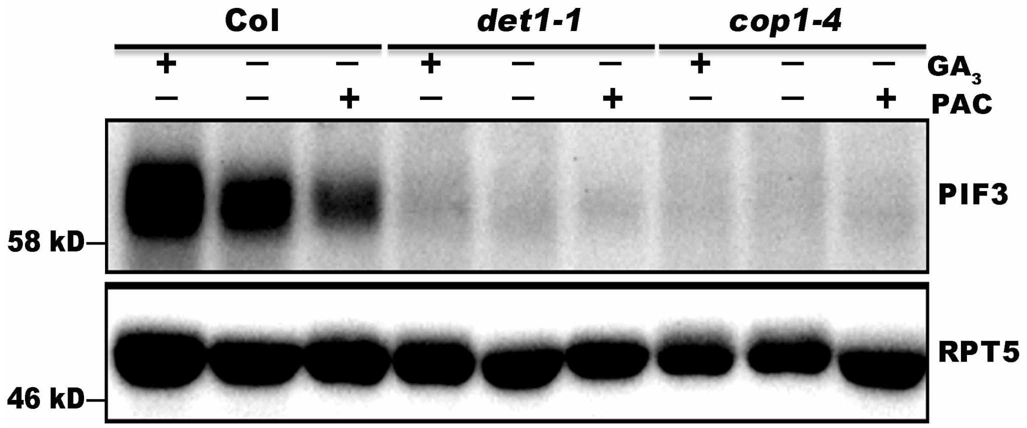

Supplementary Figure 4. Treatments of GA and PAC could not modulate PIF3 abundance in the mutants of *COP1* and *DET1*. Endogenous PIF3 protein levels were checked in Col, *det1-1*, and *cop1-4* seedlings grown on medium containing 10 μM GA_3 or 0.5 μM PAC in the dark for 4 days. RPT5 was used as a loading control.

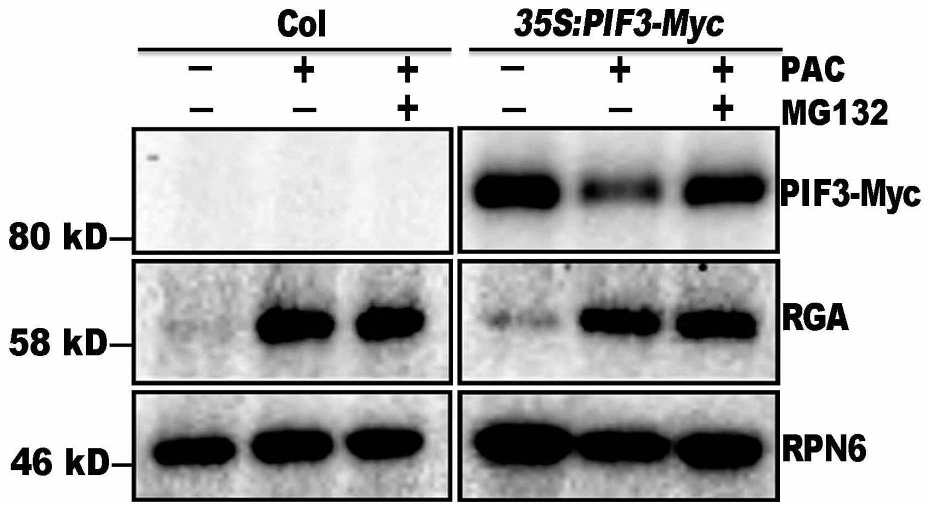

Supplementary Figure 5. RGA and PIF3-Myc protein levels in the seedlings used for ChIP analysis in Figure 7a. 4-day-old dark-grown seedlings were treated with PAC or PAC plus MG132. After the fixation (1% formaldehyde, 15 min) and quenching of formaldehyde (2 M glycine, 5 min), total proteins from the same seedlings collected for ChIP assay were analyzed by immunoblot. RPN6 was used as a loading control.

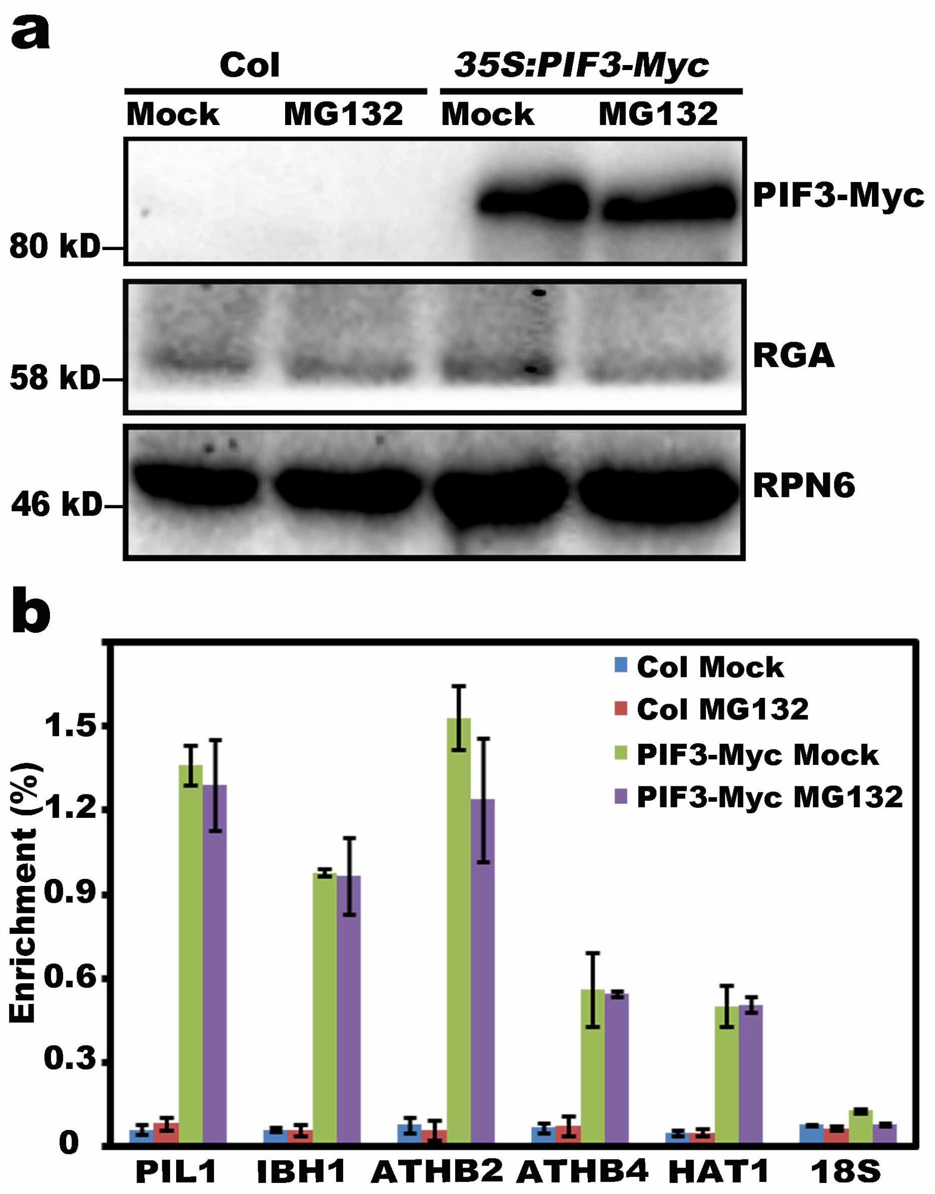

Supplementary Figure 6. ChIP analysis of the binding of PIF3 to its target genes with or without MG132 treatment. 4-day-old dark-grown Col and *35S:PIF3-Myc* seedlings were collected and treated with DMSO or 100 μM MG132 for 4 h. **(a)** After the fixation (1% formaldehyde, 15 min) and quenching of formaldehyde (2 M glycine, 5 min), total proteins from the same seedlings collected for ChIP assay were analyzed by immunoblot. RPN6 was used as a loading control. **(b)** ChIP-qPCR analysis of the binding of PIF3-Myc to PIF3's target genes.18S rDNA was used as a non-binding control. The data was calculated from three biological replicates.

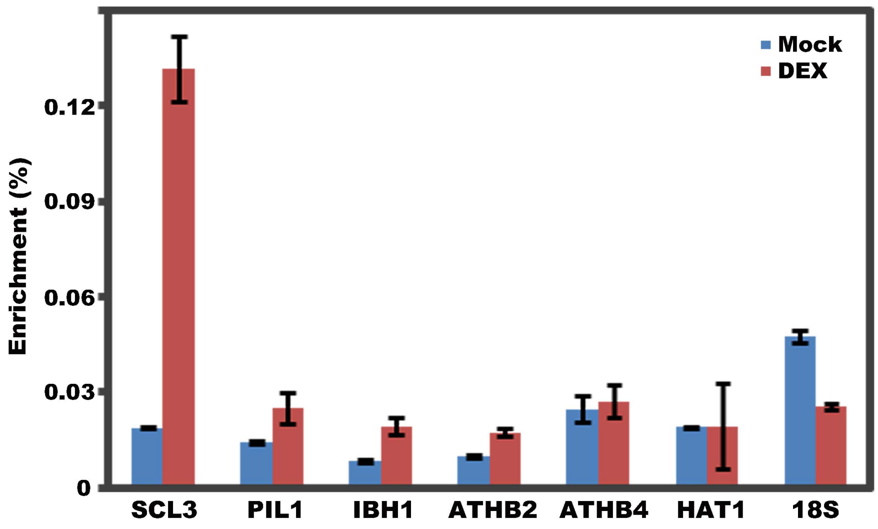

Supplementary Figure 7. ChIP analysis of the binding of RGAΔ17-HA to the target genes of PIF3. 4-day-old dark-grown RGAΔ17-HA seedlings were collected and infiltrated with or without 10 μM DEX for 24 h. 18S rDNA was used as a non-binding control. The data was calculated from two biological replicates.

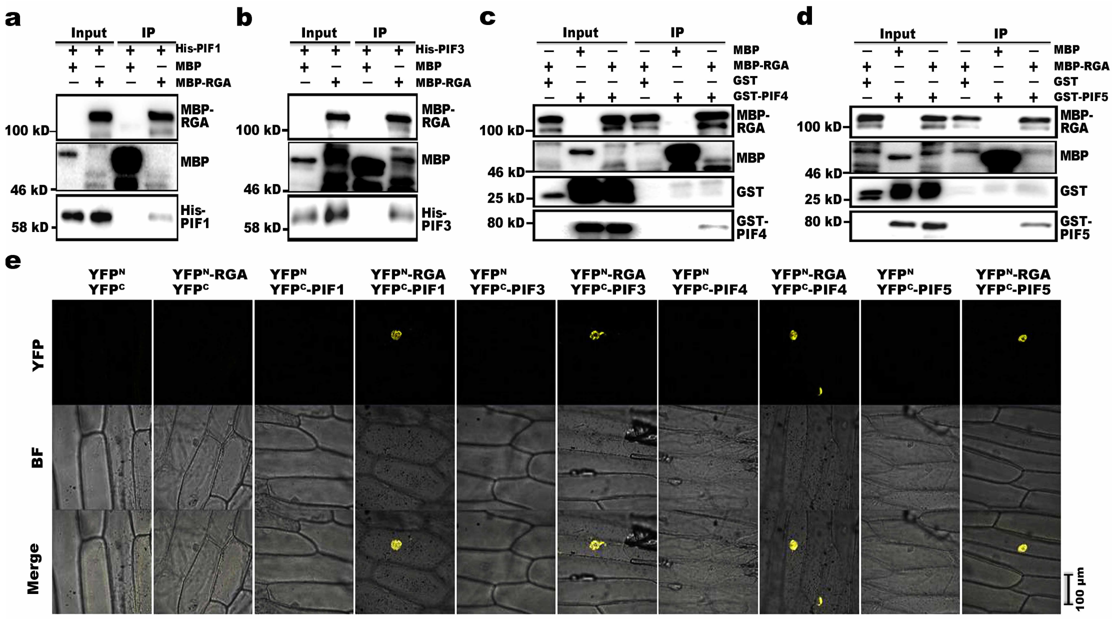

Supplementary Figure 8. RGA interacts with PIFs both *in vitro* and *in vivo*. (a-b) MBP-RGA can pull down His-PIF1 and His-PIF3 *in vitro*. Recombinant MBP-RGA or MBP was incubated with either PIF1 or PIF3 fused to His, and the precipitated fractions were analyzed with anti-MBP and anti-His antibodies. **(c-d)** MBP-RGA can pull down GST-PIF4 and GST-PIF5 *in vitro*. Recombinant MBP-RGA or MBP was incubated with either PIF4 or PIF5 fused to GST, or GST itself, and the precipitated fractions were analyzed with anti-MBP and anti-GST antibodies. **(e)** BiFC analysis of the interactions between RGA and PIFs. YFP^N-RGA (N-terminal fragment of yellow fluorescent protein fused with RGA) and YFP^C-PIFs (C-terminal fragment of yellow fluorescent protein fused with each of the four PIF proteins) were transiently transformed into onion epidermal cells. YFP fluorescence images (upper panel), bright field view images (middle panel), and fluorescence images merged with bright field view images (lower panel) were shown. All images were under the same magnification.

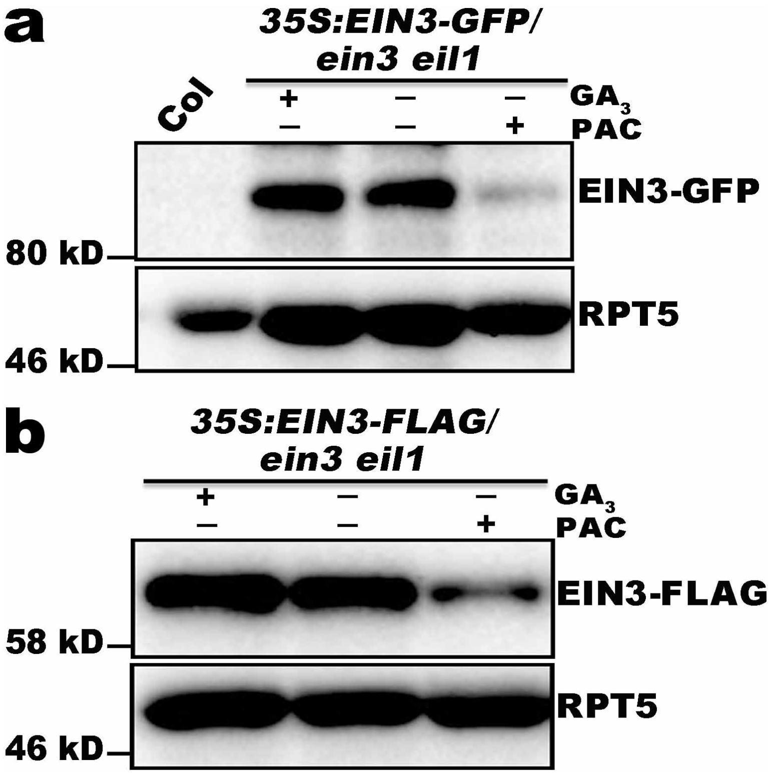

Supplementary Figure 9. DELLAs negatively regulate EIN3 protein abundance upon PAC treatment in darkness. Effects of GA_3 or PAC treatments on EIN3-GFP **(a)** and EIN3-Flag **(b)** protein levels. The seedlings were grown in the medium with indicated supplements (10 μM GA_3 or 0.5 μM PAC) in the dark, and total proteins were analyzed by immunoblots using anti-GFP, anti-Flag, and anti-RPT5. RPT5 was used as a loading control.

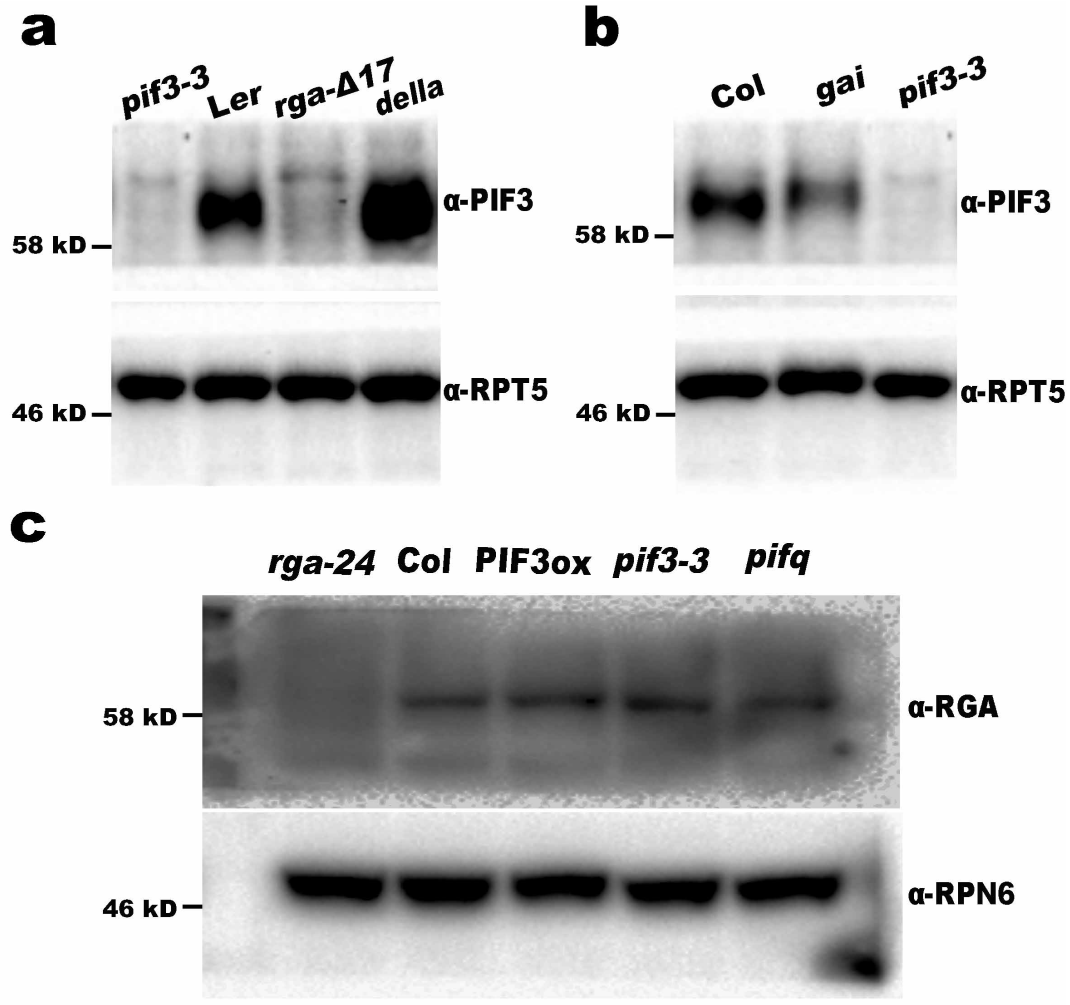

Supplementary Figure 10. Full scan of immunoblots in Figures 1b (a), 1c (b) and 1d (c). Labels are the same as in figures.

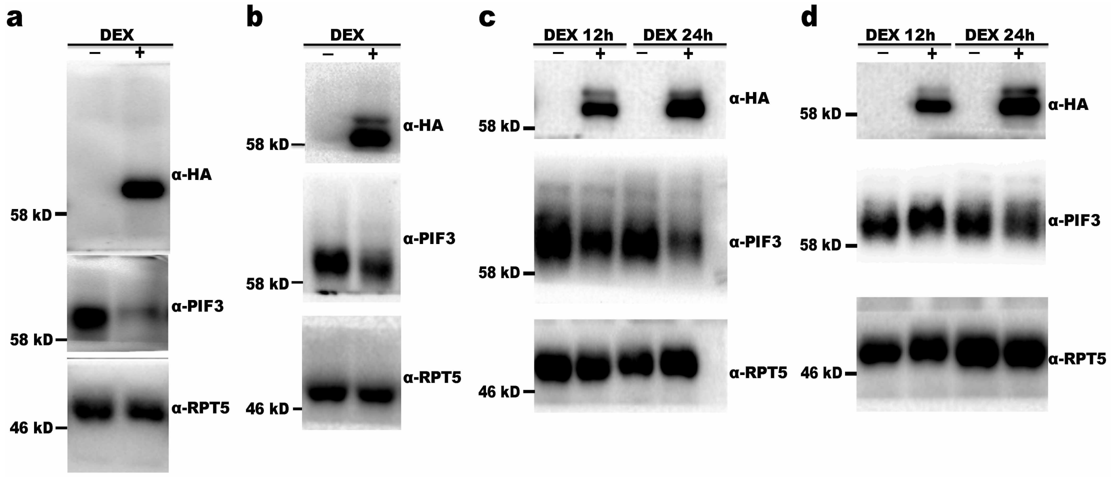

Supplementary Figure 11. Full scan of immunoblots in Figures 2c (a), 2d (b), 2e (c) and 2f (d). Labels are the same as in figures.

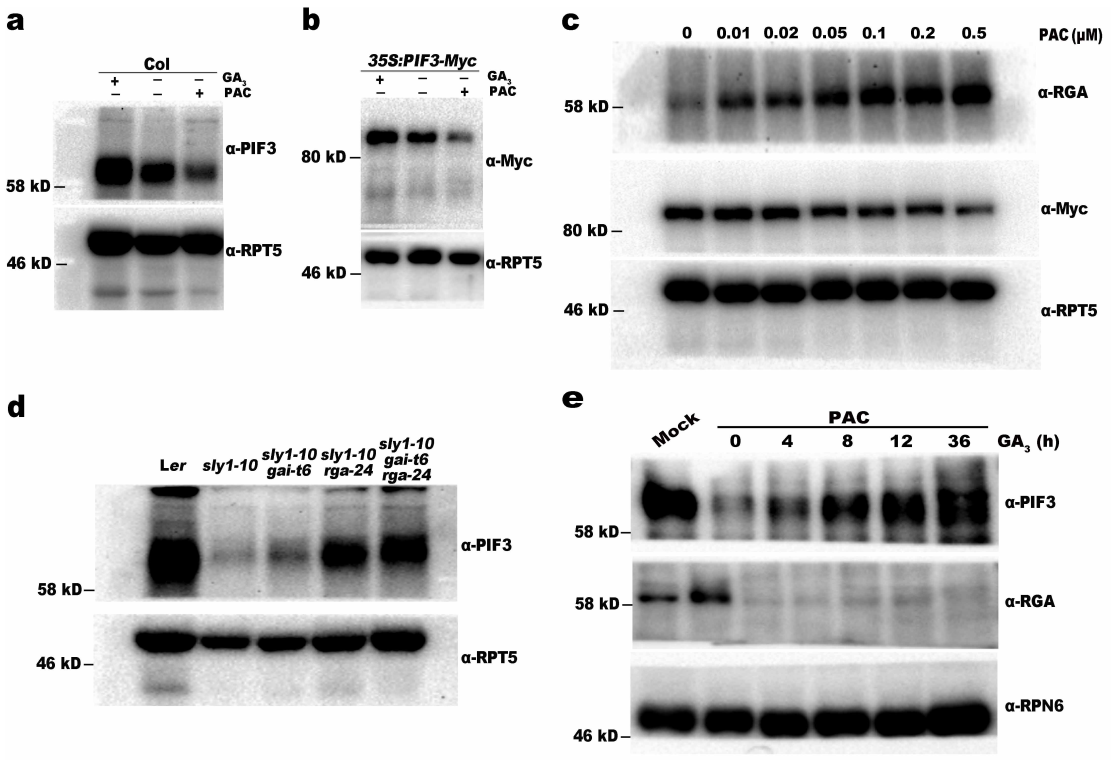

Supplementary Figure 12. Full scan of immunoblots in Figures 3b (a), 3c (b), 3e (c), 3h (d) and 3i (e). Labels are the same as in figures.

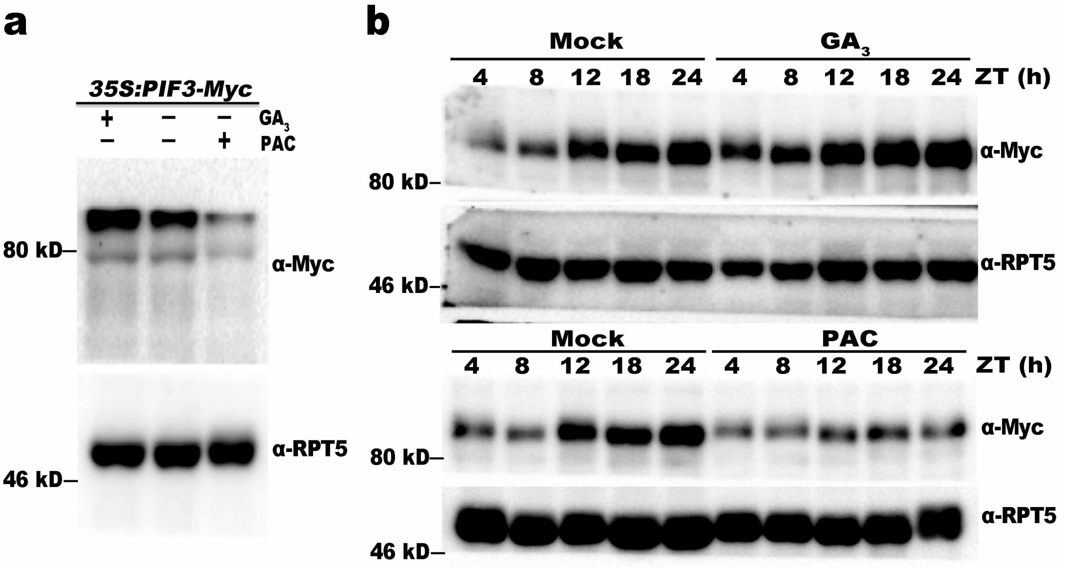

Supplementary Figure 13. Full scan of immunoblots in Figures 4b (a) and 4c (b). Labels are the same as in figures.

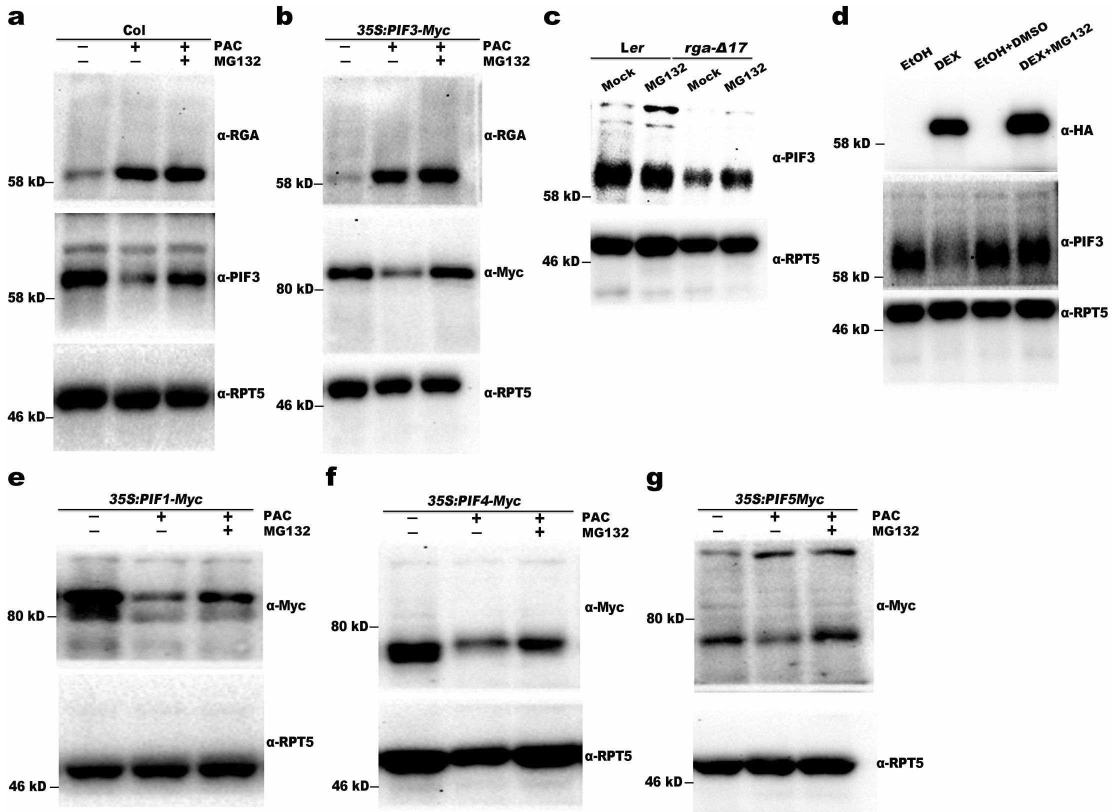

Supplementary Figure 14. Full scan of immunoblots in Figures 5a (a), 5b (b), 5c (c), 5d (d), 5e (e), 5f (f) and 5g (g). Labels are the same as in figures.

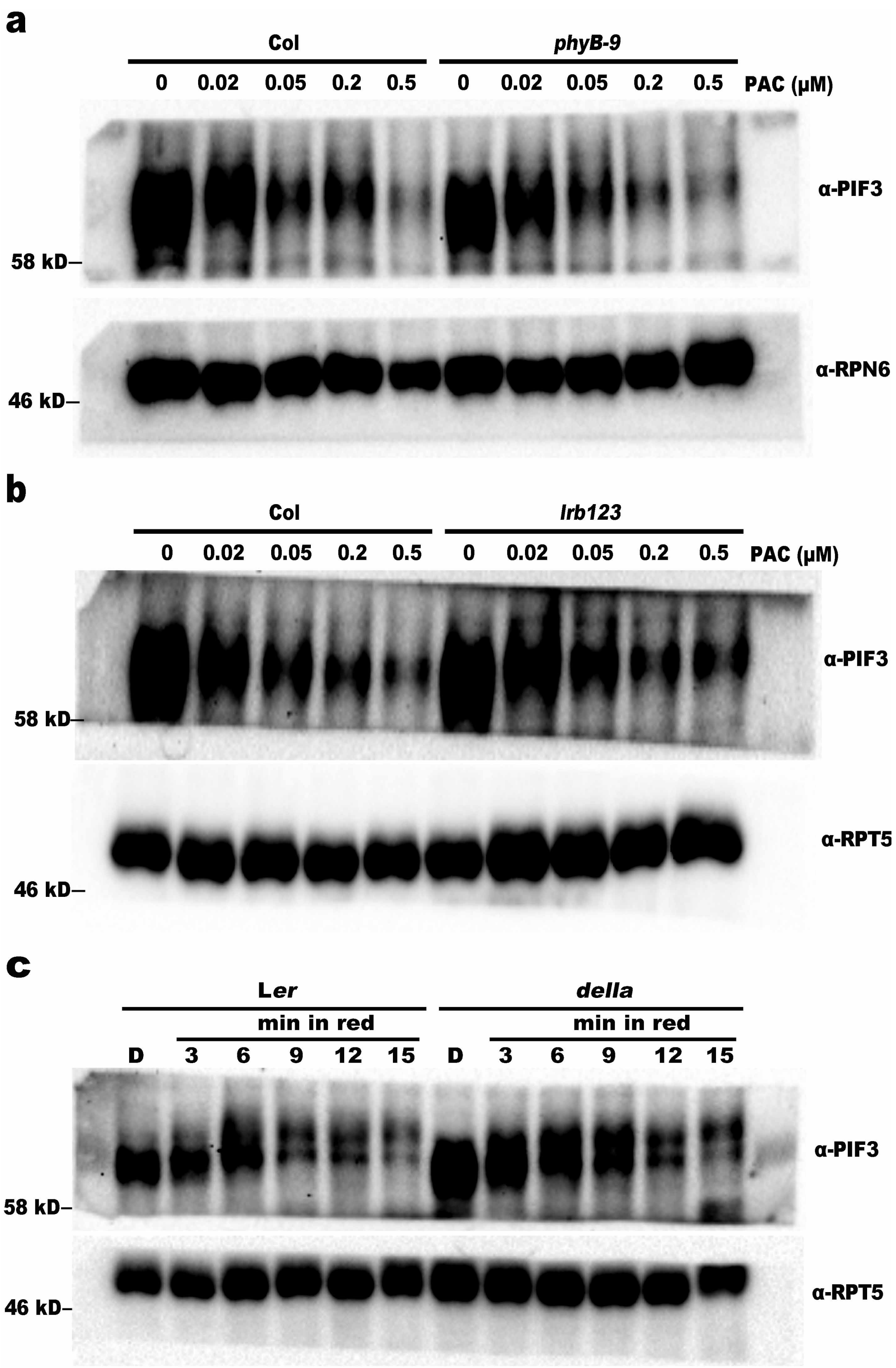

Supplementary Figure 15. Full scan of immunoblots in Figures 6a (a), 6c (b) and 6e(c). Labels are the same as in figures.

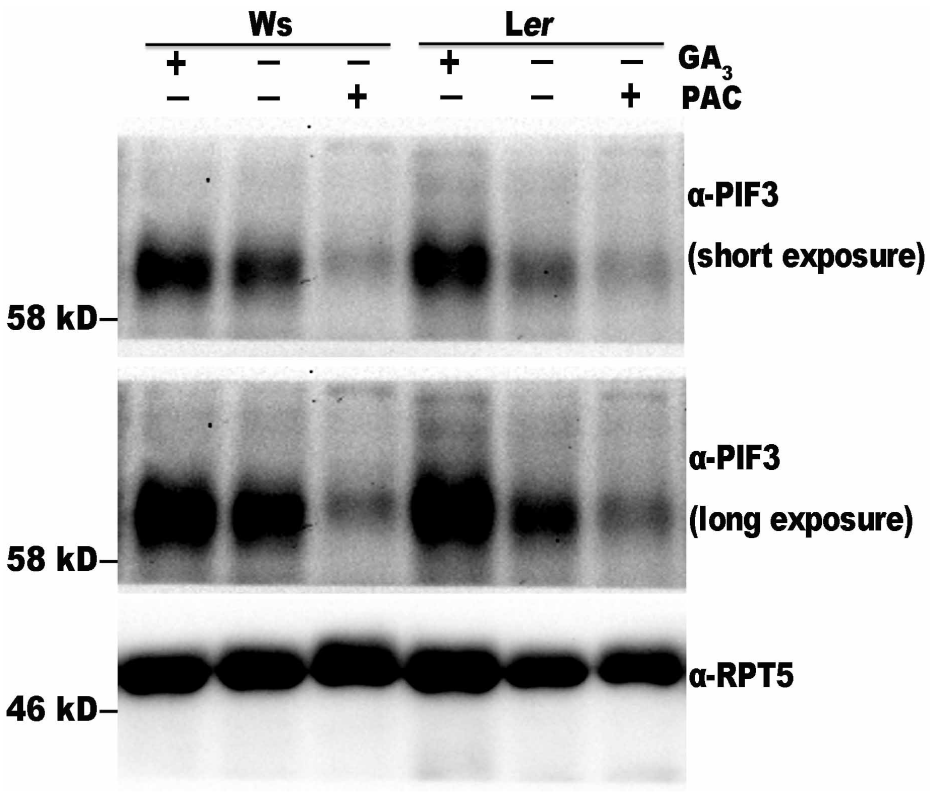

Supplementary Figure 16. Full scan of immunoblots in Supplementary Figure 2. Labels are the same as in the figure.

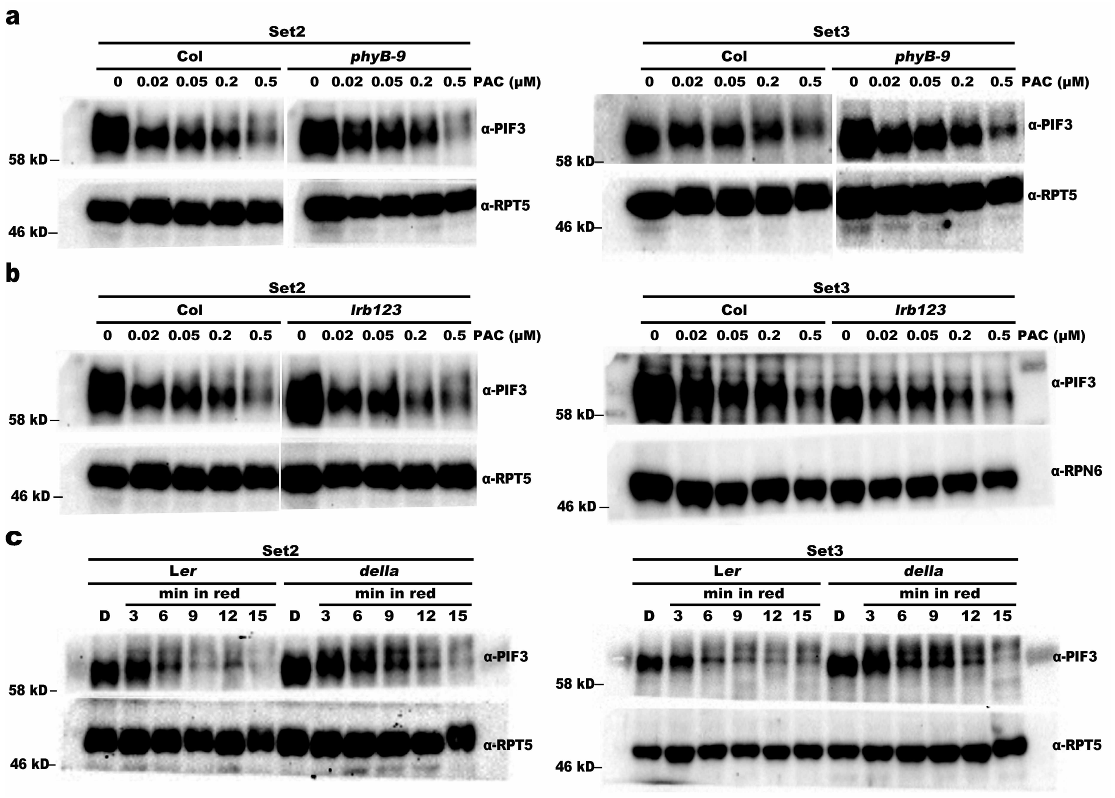

Supplementary Figure 17. Full scan of immunoblots in Supplementary Figure 3. Labels are the same as in figures.

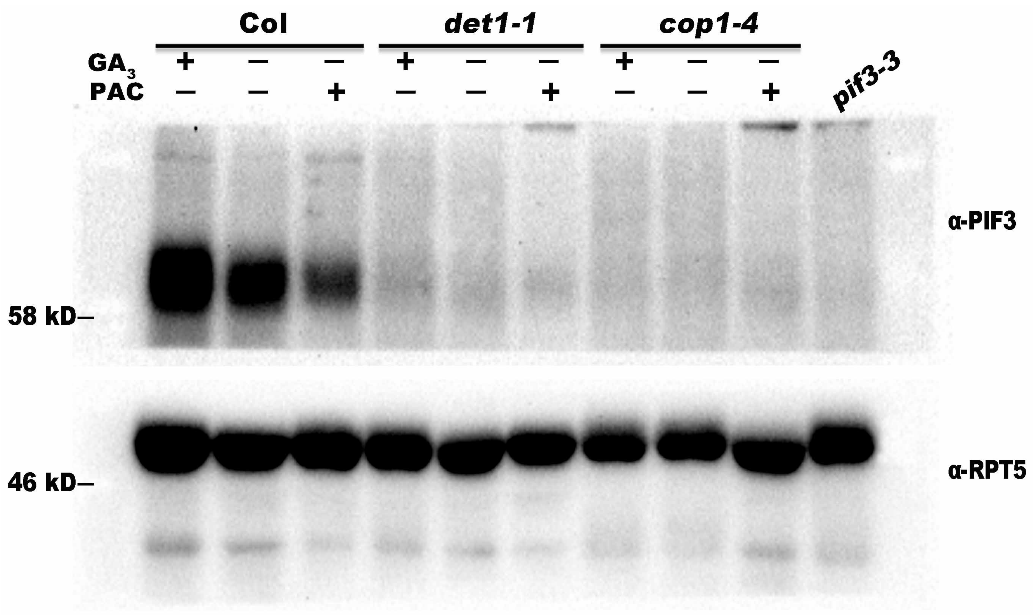

Supplementary Figure 18. Full scan of immunoblots in Supplementary Figure 4. Labels are the same as in the figure.

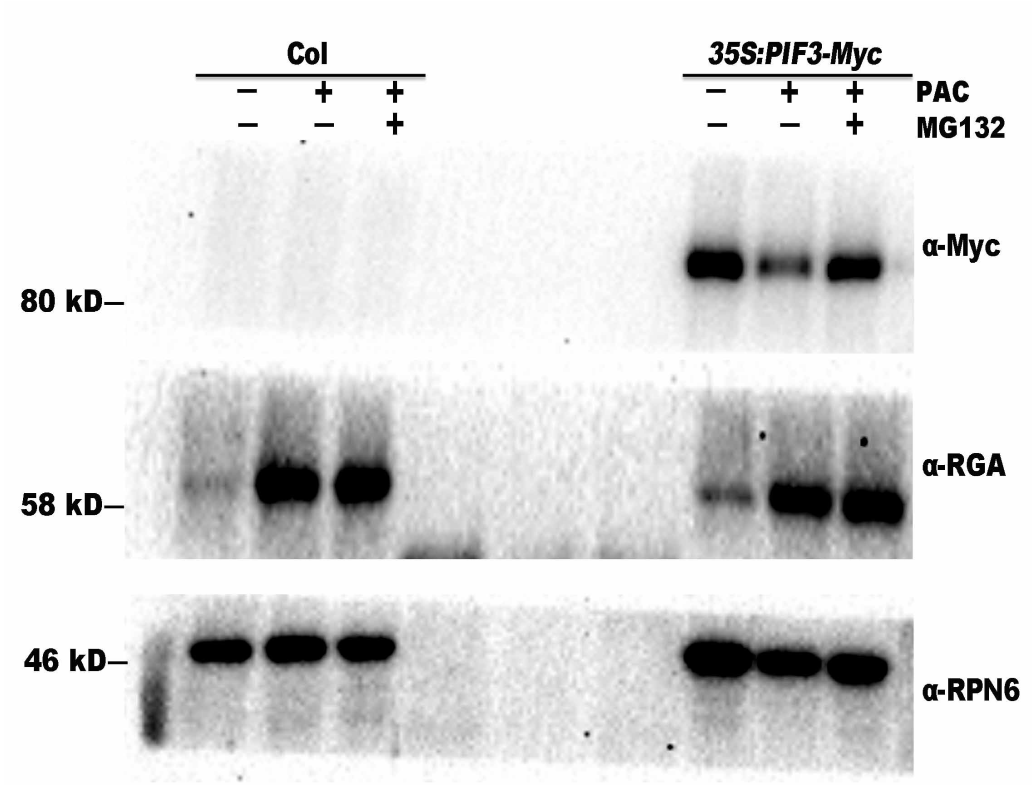

Supplementary Figure 19. Full scan of immunoblots in Supplementary Figure 5. Labels are the same as in the figure.

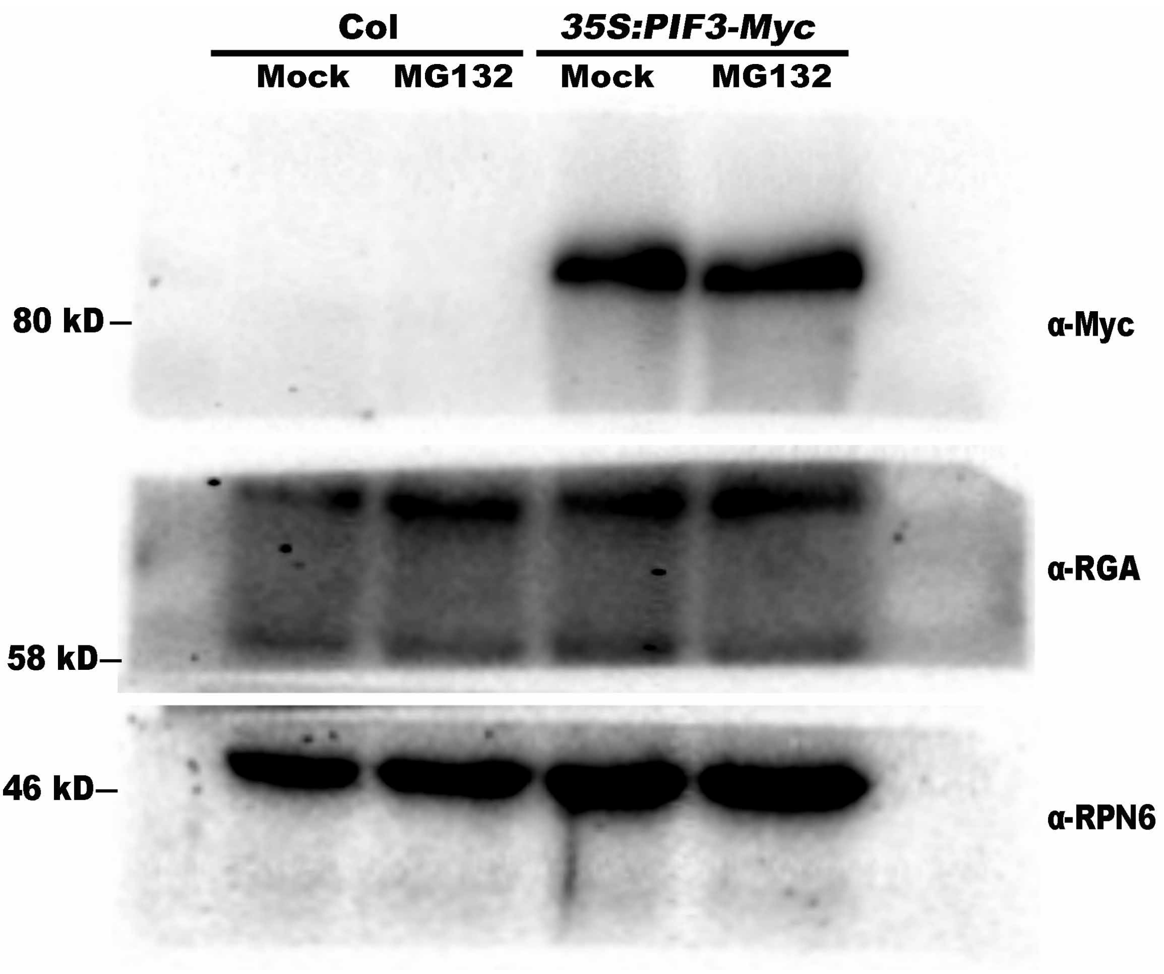

Supplementary Figure 20. Full scan of immunoblots in Supplementary Figure 6. Labels are the same as in the figure.

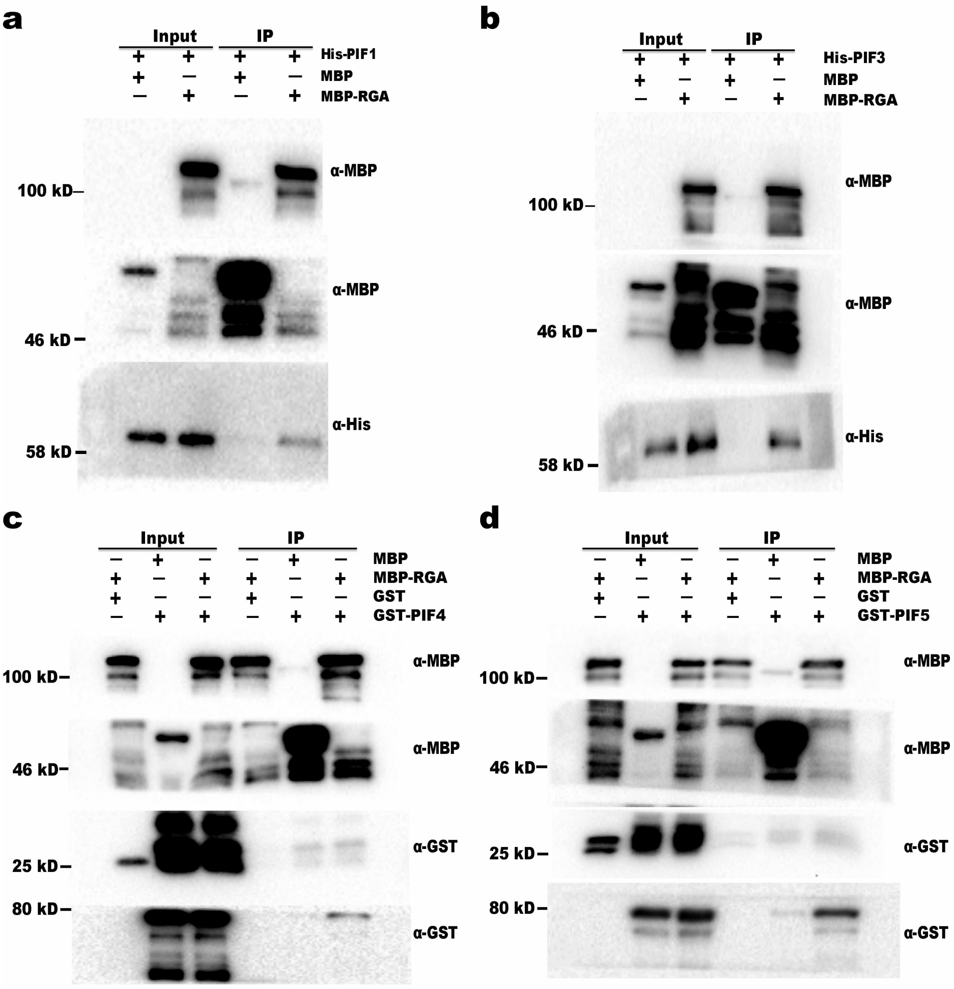

Supplementary Figure 21. Full scan of immunoblots in Supplementary Figure 8. Labels are the same as in the figure.

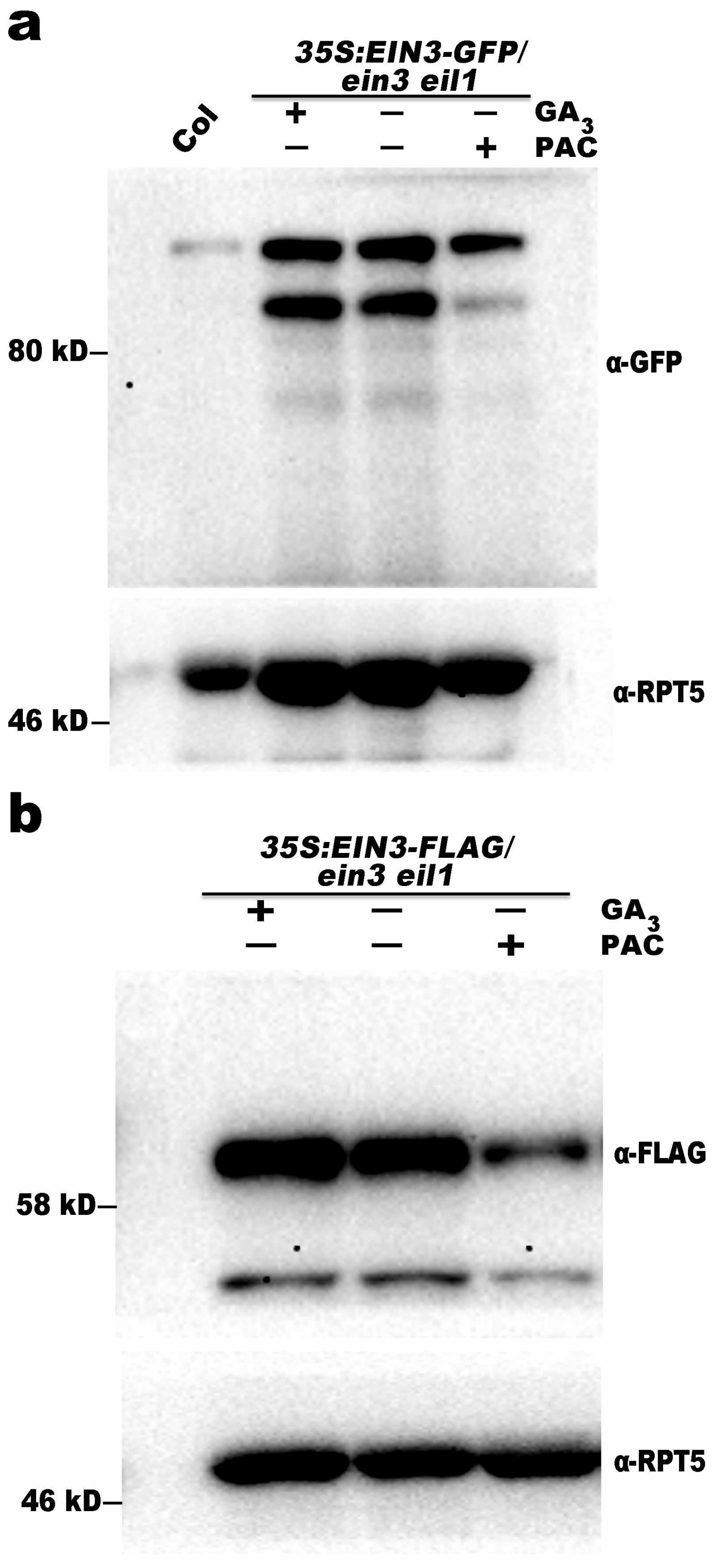

Supplementary Figure 22. Full scan of immunoblots in Supplementary Figure 9. Labels are the same as in the figure.

Supplementary Table1. T-test analysis of the hypocotyl lengths in Fig. 3a (Mock vs GA_3)

Seedlings No.	Mock (cm)	GA_3 treatment (cm)
1	1.48	1.619
2	1.491	1.622
3	1.497	1.622
4	1.506	1.637
5	1.509	1.638
6	1.511	1.644
7	1.524	1.657
8	1.525	1.661
9	1.533	1.673
10	1.551	1.688
11	1.551	1.69
12	1.554	1.701
13	1.567	1.707
14	1.575	1.709
15	1.577	1.71
16	1.579	1.727
17	1.583	1.732
18	1.587	1.744
19	1.609	1.744
20	1.611	1.745
21	1.624	1.758
22		1.772
23		1.773
24		1.778

Unpaired t test	
P value	< 0.0001
P value summary	***
Are means signif. different? (P < 0.05)	Yes
One- or two-tailed P value?	Two-tailed
t, df	t=10.43 df=43
How big is the difference?	
Mean ± SEM of Mock	1.550 ± 0.009201 N=21
Mean ± SEM of GA_3	1.698 ± 0.01058 N=24
Difference between means	-0.1482 ± 0.01422
95% confidence interval	-0.1769 to -0.1196
R squared	0.7166

Supplementary Table 2. List of primers used in this study

Primers	Sequence 5'-3'
pBSK-GAIΔ17-F	TCCCCCCGGGATGAAGAGAGATCATCATCATCATCAT
pBSK-GAIΔ17-R	AAAACTGCAGATTGGTGGAGAGTTTCCAAGC
pBSK-RGAΔ17-F	TCCCCCCGGGATGAAGAGAGATCATCACCAAT
pBSK-RGAΔ17-R	AAAACTGCAGGTACGCCGCCGTCGA
pTA7002-GAIΔ17-HA-F	ACGCGTCGACATGAAGAGAGATCATCATCATCATCAT
pTA7002-RGAΔ17-HA-F	ACGCGTCGACATGAAGAGAGATCATCACCAAT
pTA7002-GAIΔ17/RGAΔ17-HA-R	GGACTAGTAAGCTTGATCCCGGGGGAG
qPP2A-F	TATCGGATGACGATTCTTCGTGCAG
qPP2A-R	GCTTGGTCGACTATCGGAATGAGAG
qPIF3-F	ATTTTCCCACACCAGCTCCACAAC
qPIF3-R	GCTCAAGACAGGAACCCTTCTCCA
YFP^N-RGA-F	GGACTAGTATGAAGAGAGATCATCACCAAT
YFP^N-RGA-R	CGGGATCC TCAGTACGCCGCCGTCGA
PIL1-CHIP-F	ATAACACAAAGGGGTGGATG
PIL1-CHIP-R	TAAATGGGACCCACAATTAG
IBH1-CHIP-F	GAGAGAAAGGAAAGTGGAGGTGGGT
IBH1-CHIP-R	GTAGAGTAGGTCCACTAATGGGCCA
ATHB2-CHIP-F	ATTTGACGGACACACCTTTC
ATHB2-CHIP-R	ACTAGTTAATAAAGCGGGACC
ATHB4-CHIP-F	TGAAGCGTGTGAATGGTGTGGGAG
ATHB4-CHIP-R	GCCGCACGAGTGTGGTCACTG
HAT-CHIP-F	TGTCGGCGCGTGAGGAAACA
HAT-CHIP-R	GGGCAGGTGGGTCATGTCACG

SCL3-CHIP-F	GCCTCAGCCTCATCTCTTTT
SCL3-CHIP-R	GGAATCATGACTATATATTTCTACATCA
18S-CHIP-F	GCTAACTAGCTACGTGGAGG
18S-CHIP-R	CATCTAAGGGCATCACAGAC
